# Cold-press sintering and particle-size engineering of Bi_0.3_Sb_1.7_Te_3_ for enhanced thermoelectric performance

**DOI:** 10.1016/j.isci.2026.115544

**Published:** 2026-03-31

**Authors:** Ruifeng Xiong, Jeremy Lemoigne, Aran Rafferty, Franck Gascoin, Amir Pakdel

**Affiliations:** 1 School of Engineering, Trinity College Dublin, The University of Dublin, D02PN40 Dublin, Ireland; 2 Laboratoire CRISMAT, ENSICAEN, UNICAEN, CNRS Normandie Université (UMR 6508), 14280 Caen, France; 3 Advanced Materials and BioEngineering Research Centre (AMBER), Trinity College Dublin, The University of Dublin, D02PN40 Dublin, Ireland

**Keywords:** Engineering, Materials science, Physics

## Abstract

Microstructural engineering is a crucial approach to improving the conversion efficiency of thermoelectric materials and enabling their broad application. In this study, Bi_0.3_Sb_1.7_Te_3_ samples were fabricated via cold pressing, hot pressing, and combined cold/hot pressing, each followed by vacuum sintering, allowing for a systematic comparison of the effect of processing on thermoelectric properties. The cold-press sintered sample exhibited excellent Seebeck coefficient and electrical conductivity. Furthermore, combining particle size control through ball milling and centrifugation with the cold-press sintering process significantly enhanced the TE performance. The sample with an average particle size of ∼200 nm achieved a high *ZT* of 1.18 at 300 K, attributed to the enhancement of the Seebeck coefficient by filtering of low energy charge carriers and the reduction of thermal conductivity through interface scattering. These findings highlight the pivotal role of microstructural engineering through powder processing in optimizing the near-room-temperature performance of Bi_0.3_Sb_1.7_Te_3_, providing guidance for further enhancement of p-type Bi-Sb-Te thermoelectrics.

## Highlights

• Bi_0.3_Sb_1.7_Te_3_ are consolidated via cold/hot pressing and cold-/hot-press sintering methods
• Cold-press sintering yielded optimal thermoelectric performance with a *ZT* of 1.05 at 325 K
• Nano Bi_0.3_Sb_1.7_Te_3_ powders further improved the TE efficiency and yielded a *ZT* of 1.18
• Microstructural engineering particularly affected the *S* and *K* due to increased scattering

## Graphical abstract

**Figure:**
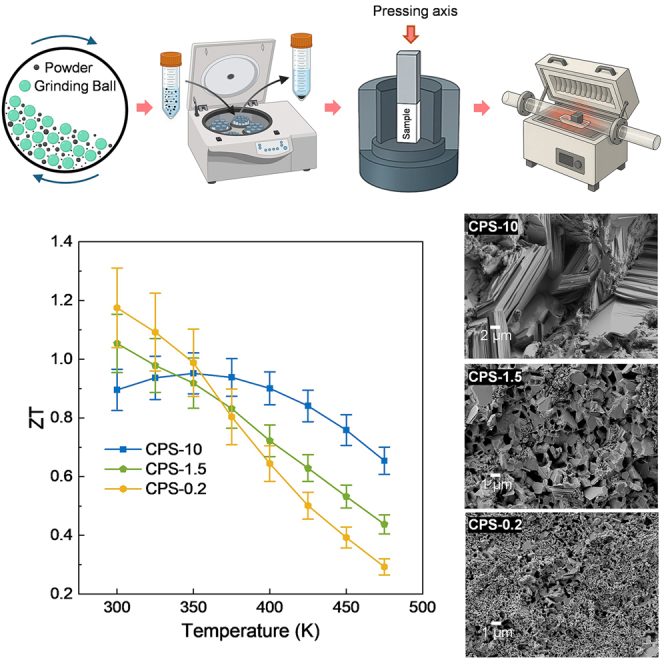

## Introduction

The escalating environmental impact of fossil fuel overconsumption has highlighted the urgent need for sustainable alternatives to conventional energy sources. Among the various emerging approaches, thermoelectric (TE) materials and devices have attracted considerable attention because they can directly convert heat into electricity in a solid-state, noise-free, and environmentally friendly manner, enabling energy recovery from waste heat. TE technologies offer unique advantages for maintenance-free energy harvesting, particularly in scenarios where waste heat is abundant but difficult to access using conventional energy conversion systems, such as in industrial processes, electronics cooling, and wearable self-powered sensing devices.^1^ The efficiency of TE materials is typically characterized by the dimensionless figure of merit *ZT*, defined as $ZT=\frac{S^{2}\sigma }{K}T$, where *S* is the Seebeck coefficient, *σ* is electrical conductivity, *K* is thermal conductivity, and *T* is the absolute temperature. The numerator, known as the thermoelectric power factor (*S*^2^*σ*), quantifies the material’s capability to generate electrical power.^2^ With growing emphasis on energy efficiency and environmental protection, improving *ZT* and optimizing fabrication methods have become major research priorities. However, high material-level *ZT* alone does not necessarily guarantee superior device-level performance or large-scale applicability. Optimizing fabrication routes and microstructural stability is equally critical for achieving reliable device-level performance. In fact, processing scalability, mechanical robustness, long-term thermal stability, and compatibility with module fabrication also play essential roles in determining the practical impact of TE materials.^3^ In this context, materials that exhibit strong TE performance near room temperature are of great interest, as they can be used to power low-energy electronic devices and broaden the range of practical TE applications, including Internet-of-Things (IoT) sensors, wearable electronics, and localized waste-heat recovery systems, where scalability, and fabrication simplicity can be more advantageous than record-high ZT values achieved under highly specialized conditions.^4^^,^^5^

Bismuth telluride-based compounds are among the most effective TE materials operating near room temperature, with p-type Bi-Sb-Te alloys particularly demonstrating excellent performance and wide practical use.^6^ Achieving high TE efficiency in these materials requires a synergistic balance between high electrical conductivity and low thermal conductivity. However, this optimization is constrained by the Wiedemann-Franz law, which describes direct proportionality between electrical and thermal conductivity, making simultaneous enhancement of electrical conductivity and reduction of thermal conductivity inherently challenging.^7^ To address this, low-dimensional structural engineering strategies have been widely employed to decouple these transport properties and improve overall TE performance. Two main approaches have been explored: (1) introducing secondary-phase micro- or nano-particles into the matrix and (2) processing fine micro- or nano-scale powders into bulk materials with refined grain structures. Both methods enhance phonon scattering at interfaces, thus reducing lattice thermal conductivity, while minimally affecting electrical transport due to the shorter mean free path of electrons compared to phonons.^8^^,^^9^ Recent studies have demonstrated that embedding hetero-inclusions such as Sb_2_O_3_,^10^ MnO_2_,^11^ PbTe,^12^ Cu, C, g-C_3_N_4_, Ga/In, FeTe_2_, Zn, Sn, Pb, and C_60_^13^ into p-type Bi-Sb-Te matrices can significantly enhance the *ZT*. Additionally, reducing the particle size by using ultrafine powders has also proven effective in enhancing TE performance of state-of-the-art p-type Bi-Sb-Te alloys, including Bi_0.3_Sb_1.7_Te_3_, Bi_0.4_Sb_1.6_Te_3_, and Bi_0.5_Sb_1.5_Te_3_.^10^^,^^13^^,^^14^^,^^15^

A wide range of fabrication strategies have been adapted to prepare micro- and nano-structured bismuth telluride-based TE materials. These include top-down approaches such as ball milling,^10^ melt spinning,^16^ and gas atomization,^17^ as well as bottom-up methods like mechanical alloying,^14^ sol-gel processing,^15^ thermal decomposition,^18^ microwave-assisted hydro/solvothermal synthesis,^16^ wet chemical techniques,^16^ electrochemical deposition,^19^ and various vapor deposition methods.^20^ Among them, ball milling stands out for its scalability, simplicity, and ability to rapidly generate fine powders from bulk ingots. To convert these powders into dense bulk samples with enhanced mechanical and TE performance, diverse compaction and sintering techniques have been employed. These include cold pressing (CP),^21^ cold pressing followed by sintering (CPS),^22^ hot pressing (HP),^16^ spark plasma sintering (SPS),^15^ cold/hot extrusion,^16^ hot forging,^23^ cold/hot isostatic pressing,^24^ microwave sintering,^25^ laser sintering,^26^ and the recently developed liquid-phase-assisted or “cold sintering” process.^27^^,^^28^ These densification routes often lead to microstructures with high densities of lattice imperfections, including grain boundaries and dislocations, which are beneficial for enhancing phonon scattering and reducing thermal conductivity, thereby improving the overall TE efficiency. Furthermore, from this device-oriented perspective, there is a strong need to systematically evaluate how different powder consolidation and densification routes influence not only TE properties but also processing robustness and suitability for scalable TE device fabrication.

In this work, the role of microstructural engineering in optimizing the TE performance of p-type Bi_0.3_Sb_1.7_Te_3_ was systematically explored. Coarse powders were first obtained via manual grinding of bulk ingots. Then, four consolidation methods were applied to fabricate bulk samples: CP, CPS, HP, and hot pressing followed by sintering (HPS). The impact of each densification approach on the Seebeck coefficient, electrical conductivity, and thermal conductivity was correlated with the resulting microstructural features. By systematically comparing these processing routes, this study aims to bridge the gap between laboratory-scale performance optimization and scalable, energy-efficient fabrication strategies that are more relevant for practical TE devices. To further investigate the influence of particle size, ball milling combined with centrifugal classification was employed to produce powders with controlled particle sizes (∼10.5 μm, ∼1.5 μm, and ∼200 nm). These graded powders were then compacted using an optimized cold-press sintering technique. The effects of particle size refinement and enhanced interface boundary density on charge and phonon transport were comprehensively analyzed, offering insights into the structure-property relationships that govern TE performance. The findings of this work provide guidance for designing TE materials and processing routes that balance performance, scalability, and sustainability, thereby supporting the development of practical TE devices for real-world energy harvesting applications.

## Results and discussion

### Effect of consolidation process on Bi_0.3_Sb_1.7_Te_3_ properties

#### Microstructural and chemical characterization

**Figure 1:**
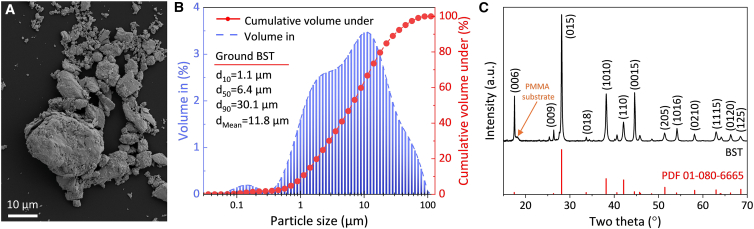
Characterization of manually ground BST powder (A) SEM image. Scale bars represent 10 μm. (B) Particle size distribution. (C) XRD pattern.

As outlined in the “materials and methods” section, p-type Bi_0.3_Sb_1.7_Te_3_ (hereafter referred to as BST) was manually ground into granular powder. A representative SEM image of the ground BST powder is shown in Figure 1A, and the corresponding particle size distribution is presented in Figure 1B. The particles exhibited irregular morphologies with a broad and non-uniform size distribution, ranging from ∼60 nm to ∼100 μm, with a volume-weighted mean particle diameter (D[4,3]) of ∼11.8 μm. To quantify the particle size distribution, the cumulative distribution points d_10_, d_50_, and d_90_ were determined. These parameters correspond to the particle sizes below which 10%, 50%, and 90% of the sample’s particles were smaller than the specified value, respectively. As shown in Figure 1B, the d_10_, d_50_, and d_90_ were 1.1, 6.4, and 30.1 μm, respectively.

The X-ray diffraction (XRD) pattern of the BST powder is shown in Figure 1C. The observed diffraction peaks correspond well with the reference pattern for Bi_0.3_Sb_1.7_Te_3_ (PDF# 01-080-6665), confirming a rhombohedral crystal structure with space group R-3m. A small peak was identified in the shoulder of the BST (006) peak, arising from the diffraction contribution of the PMMA substrate. Within the detection limits of the instrument, no secondary phases were identified, and no noticeable peak shifts were observed, indicating that the nominal composition was preserved.

**Figure 2:**
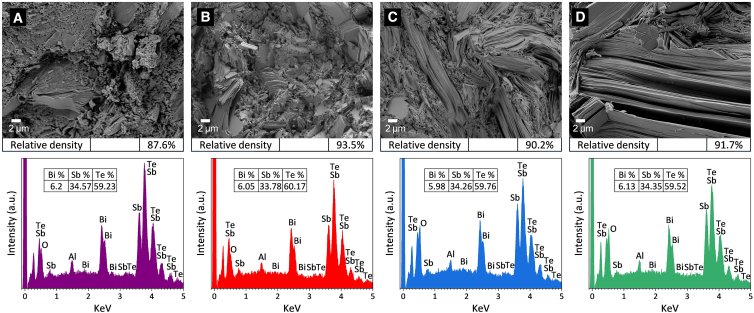
Cross-sectional SEM images and EDS analysis of BST samples (A) Cold-pressed. Scale bars represent 2 μm. (B) Cold-pressed and sintered. Scale bars represent 2 μm. (C) Hot-pressed. Scale bars represent 2 μm. (D) Hot-pressed and sintered. Scale bars represent 2 μm.

The ground BST powders were consolidated into bulk samples using four different processing conditions: CP at ambient temperature, HP at 693 K, CPS at 693 K, and HPS at 693 K. Additional procedural details can be found in the “materials and methods” section. Figure 2 presents SEM cross-sectional images of the samples prepared by these fabrication routes. As shown in Figures 2A and 2B, both CP and CPS samples exhibited granular and layered structures with broad size distributions, including a mixture of micro- and nano-scale particles. In the CP sample, the particle sizes resemble those of the original powder, and the particles are loosely connected, resulting in large interparticle void spaces and a relative density of 87.6% (more details in Data S1). After sintering, coarser particles were observed, and the relative density increased to 93.5%, consistent with improved particle consolidation. In contrast, the HP and HPS samples shown in Figures 2C and 2D displayed a more coarsened and textured lamellar microstructure. This indicates that many of the initial particles merged into large, plate-like grains during these processes, and formed relatively continuous layered regions. The relative densities of the HP and HPS samples reached 90.2% and 91.7%, respectively. Furthermore, energy-dispersive X-ray spectroscopy (EDS) revealed consistent elemental compositions across all samples, characterized by the presence of Bi, Te, and Sb peaks. The corresponding quantitative analysis (atomic concentration) is provided in the inset tables of Figure 2. Additional signals from C, O, and Al were attributed to the carbon tape and aluminum sample holder. Stronger O peaks were detected in the HP and HPS samples, associated with minor surface oxidation (further details are provided in Data S2).

#### Thermoelectric characterization

**Figure 3:**
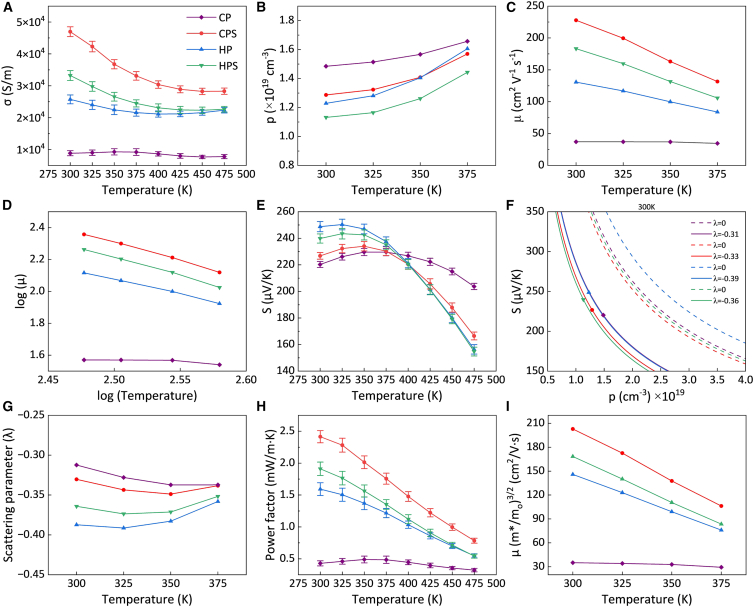
Electrical transport properties in cold-pressed, cold-pressed and sintered, hot-pressed, and hot-pressed and sintered Bi_0.3_Sb_1.7_Te_3_ samples Data are represented as mean ± SEM. (A–C) Temperature dependence of (A) electrical conductivity, (B) charge carrier concentration, and (C) charge carrier mobility. (D) Measurement of parameter *x* in the temperature dependence of mobility described as *μ* ∝ *Tx*. (E) Temperature dependence of the Seebeck coefficient. (F) Seebeck coefficient versus charge carrier concentration (Pisarenko lines) at 300 K. (G–I) Temperature dependence of (G) SPB scattering parameter, (H) power factor, and (I) weighted mobility.

Figure 3A illustrates the temperature-dependent electrical conductivity (*σ*) of p-type BST samples measured between 300 and 475 K. The *σ* of the CPS, HP, and HPS samples exhibited a decreasing trend with temperature, which is characteristic of metal-like behavior in degenerate semiconductors. On the other hand, the CP sample initially showed a slight increase in *σ* at low temperatures, followed by a decrease at higher temperatures, which may be related to an annealing effect that improves interparticle contacts at higher temperatures. Overall, *σ* was closely associated with the relative density of the samples. The significantly lower *σ* of the CP sample (8.8 × 10^3^ S m^−1^) compared to the other samples was primarily attributed to its low bulk density and smaller particle morphology (as shown in Figure 2A), which intensify interface boundary scattering of charge carriers. Here, the term interface boundary refers to inter-particle contact regions formed during powder consolidation, rather than crystallographic grain boundaries within individual particles. After sintering, the CPS sample exhibited increased bulk density, improved particle connectivity, and particle coarsening, leading to a 5.3-fold increase in *σ* at room temperature (47.0 × 10^3^ S m^−1^). In comparison, the HP and HPS samples consolidated at high temperature showed lower electrical conductivities near room temperature (25.7 × 10^3^ and 33.2 × 10^3^ S m^−1^, respectively) compared to the CPS sample (more details in Data S1). Although both HP and HPS samples developed larger particles (Figures 2C and 2D), the slight reduction in density and the minor oxidation observed during processing appears to have adversely impacted their electrical transport properties (more details in Data S2).

The *σ* of Bi_0.3_Sb_1.7_Te_3_ is governed by the hole concentration (*p*) and mobility (*μ*), expressed as *σ* = *μ*·*p*·*e*, where *e* is the elementary charge. As shown in Figure 3B, Hall effect measurements indicated that the carrier concentration of all samples increased with temperature in the range 300–375 K. This trend is consistent with thermal excitation in a narrow-bandgap system and with the progressive ionization/activation of native defect states. In BST alloys, the hole concentration is strongly influenced by the balance of intrinsic point defects, such as antisite defects and vacancies.^29^ Acceptor-type defects commonly reported in p-type BST (e.g., Bi antisite on the Te site and/or Bi vacancies) increase the hole concentration, whereas donor-type defects (e.g., Te antisite on the Bi site and/or Te vacancies) can compensate holes depending on stoichiometry and processing conditions.^30^^,^^31^ Consequently, the measured *p* reflects the net outcome of competing native defects rather than a single defect type.^32^ Among all samples, the CP sample exhibited the highest *p*, attributed to the retention of a large number of lattice defects and vacancies during CP. After high-temperature processing, the increased densification and partial healing of defects led to reduced *p* in the CPS, HP, and HPS samples compared to CP. Additionally, the slight oxidation detected in the HP and HPS samples could further contribute to the decrease in *p*. Figure 3C shows the temperature dependence of carrier mobility, indicating that for all samples, *μ* decreased with increasing temperature, consistent with enhanced carrier-phonon interactions at higher temperatures. The CP sample displayed the lowest *μ* (37.2 cm^2^ V^−1^ s^−1^), mainly because limited intergranular contact can severely restrict charge transport, resulting in reduced *σ*. After sintering, the CPS sample exhibited significantly improved *μ* owing to better particle connectivity and reduced carrier scattering at interface boundaries. At room temperature, the *μ* of the CPS sample increased by approximately 6.1 times (227.8 cm^2^ V^−1^ s^−1^) compared to the CP sample. In the HP and HPS samples, slight oxidation introduced interfacial potential barriers that mildly reduced *μ*, with room temperature values of 130.7 and 183.2 cm^2^ V^−1^ s^−1^, respectively. Therefore, it can be concluded that the enhanced *σ* in the CPS sample was primarily attributed to the significant improvement in *μ*.

The relationship between *μ* and temperature can be utilized to infer the dominant charge carrier scattering mechanisms.^33^ Based on a single parabolic band (SPB) model and a single dominant scattering process, the temperature dependence of *μ* can be described by Equation 1:

(Equation 1) $$\mu \propto T^{x}$$

where the exponent *x* indicates the scattering mechanism. Values of *x* equal to −3/2, 0, and 3/2 correspond to scattering by acoustic phonons, neutral impurities, and ionized impurities, respectively. Additionally, polar and nonpolar optical phonon scattering may lead to exponents ranging between −1/2 and −5/2.

The slope of each line in Figure 3D, obtained from log(*μ*) versus log(*T*), corresponds to the exponent *x*. For the CP sample, the slope was −0.27, suggesting that polar optical phonon and neutral impurities scattering could be dominant mechanisms. In contrast, the slopes for the CPS, HP, and HPS samples were more negative, ranging from −1.87 to −2.32. These steeper slopes imply that phonon scattering (possibly involving a combination of acoustic and optical phonon scattering) was the prevailing mechanism for samples processed at higher temperatures. Previous studies on various TE materials have also reported significant deviations from ideal theoretical exponents, especially when the material properties showed strong temperature dependence. For example, temperature-dependent *μ* behaviors following *μ*∝*T*^−2.04^ ^34^ and *μ*∝*T*^−1.94^ ^35^ have been observed in p-type Bi-Te alloys, and *μ*∝*T*^−1.63^ ^36^ in n-type Bi-Te alloys.

Figure 3E presents the temperature dependence of the *S*. For all samples, *S* initially increased with temperature, then decreased after reaching a maximum. The CP and CPS samples reached their peaks at 350 K, while the HP and HPS samples exhibited maxima at 325 K. This behavior is associated with the bipolar effect, which becomes significant at high temperatures in narrow-bandgap materials when thermally generated minority carriers begin to contribute to transport. A higher concentration and mobility of the majority carriers delay the onset of bipolar conduction to higher temperatures, whereas lower values facilitate an earlier emergence of the bipolar effect, which is consistent with the observed shift of the peak *S* temperature among the samples. The relatively higher *S* observed in the HP and HPS samples may be attributed to their reduced *p*. Moreover, since an increased acceptor-defect density in BST can shift the Fermi level deeper into the valence band, thereby reducing the *S*.^37^ This is consistent with the comparatively lower *S* observed in the CP and CPS samples. At room temperature, the *S* of the CP, CPS, HP, and HPS samples were 220, 227, 249, and 240 μV K^−1^, respectively. Furthermore, the apparent band gap (*E*_*g*_) was estimated from the temperature dependence of the *S* using the Goldsmid-Sharp relation, *E*_g_ ≈ 2*q*|*S*|_max_*T*_max_ (more details in Data S3), where *q* is the elementary electric charge, *S*_max_ and *T*_max_ denote the maximum *S* and its corresponding temperature. For all consolidation routes (CP, CPS, HP, and HPS), the extracted values were approximately *E*_g_ ≈ 0.16 ± 0.01 eV, with no systematic dependence on processing method. These results indicate that the various consolidation strategies did not significantly modify the band structure of BST, and that the observed changes in transport behavior mainly originated from microstructural effects and defect-related scattering rather than from band-gap variation.

To further clarify the influence of *p* on the *S*, a Pisarenko analysis based on the SPB model was carried out using Equation 2 (more details in Data S4):

(Equation 2) $$S=\left(1+\lambda \right)\frac{8\pi^{2}k_{B}^{2}T}{3eh^{2}}m^{*}{\left(\frac{\pi }{3p}\right)}^{2/3}$$

where *λ* is the scattering parameter, *k*_*B*_ is the Boltzmann constant, *h* is Planck’s constant, *m∗* is the density-of-states effective mass of the charge carriers, and *p* is the carrier concentration. Figure 3F presents the Pisarenko plots at room temperature constructed from Equation 2 for different values of *λ*. All four samples generally followed the expected inverse correlation between *S* and *p*. Figure 3G shows the calculated energy-dependent scattering parameters as a function of temperature. For all samples, |*λ*| first increased and then decreased, consistent with the decrease in the *S* observed in Figure 3E. This behavior also suggests that increasing the temperature lowered the effective barrier height and promoted phonon-dominated transport, leading to a slight increase in the effective |*λ*|, in agreement with the trend inferred from Figure 3D. The |*λ*| values of the HP and HPS samples were larger than those of the CP and CPS samples, indicating stronger energy-dependent scattering. This trend is consistent with the presence of slight oxidation, which is expected to form potential barriers at interface boundaries and enhance interfacial scattering, making *λ* more negative, and thereby contributing to the increased *S* and reduced *μ* in the HP and HPS samples.

The power factor *PF = σS*^2^ is commonly used to evaluate the electronic transport performance of TE materials. Figure 3H displays the temperature dependence of the *PF* for all BST samples. The *PF* generally decreased with temperature, reflecting the trade-off between variations in the *S* and *σ*. Although the CP sample exhibited a relatively low *PF* in the range of 300–475 K (0.32–0.49 mW m^−1^ K^−1^), the sintering process led to a substantial improvement of *PF*. The CPS sample exhibited the highest *PF* across the entire temperature range, with a maximum value of 2.41 mW m^−1^ K^−1^ at room temperature. This enhancement is attributed to the simultaneous increase in both *σ* and *S*. On the other hand, while the HP and HPS samples exhibited superior *S*, their lower *σ* resulted in 34% and 21% smaller *PF* values (1.59 and 1.91 mW m^−1^ K^−1^, respectively) than that of the CPS sample at room temperature.

As depicted in Figure 3I, the weighted mobility (*μ*_*w*_), defined as *μ*(*m∗/m*_0_)^*3/2*^, which integrates both *μ* and the *m∗*, was evaluated for all samples across the range 300–375 K. A consistent trend with the *PF* plot (Figure 3H) was observed, with the CPS sample exhibiting the highest weighted mobility. This result indicates that the CPS sample possessed the most favorable electronic transport characteristics for TE performance.

**Figure 4:**
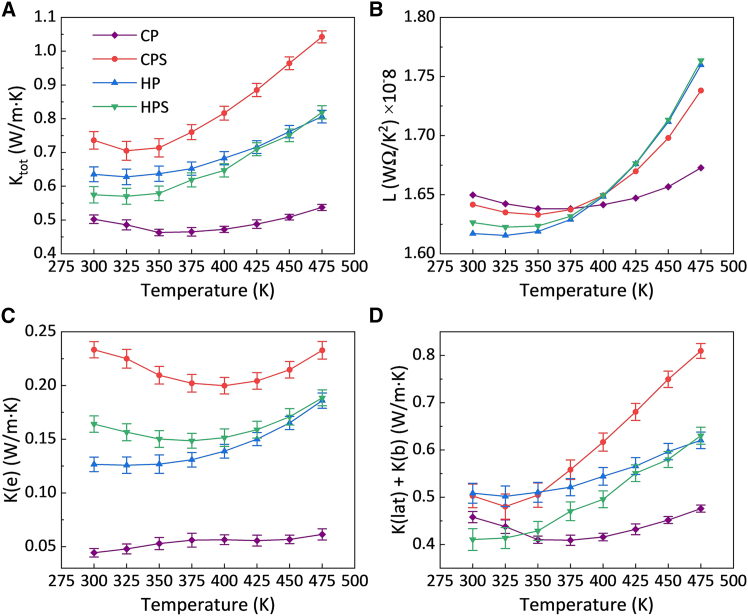
Thermal transport properties in Bi_0.3_Sb_1.7_Te_3_ samples Data are represented as mean ± SEM. (A–D) Temperature dependence of (A) total thermal conductivity, (B) Lorentz number, (C) electronic component of thermal conductivity, and (D) lattice plus bipolar components of thermal conductivity.

Figure 4 presents the thermal transport behavior of the BST samples as a function of temperature. As shown in Figure 4A, the total thermal conductivity (*K*_*tot*_) of all samples initially decreased with temperature, and after reaching a minimum point it increased. The minimum *K*_*tot*_ for the CP sample was observed at 350 K, while the lowest *K*_*tot*_ values for the CPS, HP, and HPS samples were observed at 325 K. This shift in the *K*_*tot*_ minimum toward a lower temperature suggests that the onset of bipolar conduction occurred earlier in the heat-treated samples, consistent with the *S* trends in Figure 3B. The CP sample exhibited significantly lower *K*_*tot*_ compared to the other samples at all temperatures, due to its low relative density and high density of lattice defects and interface boundaries. The lower *K*_*tot*_ values of the HP and HPS samples compared to the CPS sample could be attributed to potential barriers formed by slight oxidation, which enhanced both carrier and phonon scattering. The minimum *K*_*tot*_ values were 0.46 W m^−1^ K^−1^ at 350 K for the CP sample and 0.71, 0.63, and 0.57 W m^−1^ K^−1^ at 325 K for the CPS, HP, and HPS samples, respectively.

To further elucidate the thermal transport performance of the samples, the *K*_*tot*_ was separated into its electronic (*K*_*e*_) and a residual component dominated by lattice thermal conductivity and a possible bipolar contribution (*K*_*lat*_+*K*_*b*_) components. The electronic contribution was calculated from the Wiedemann-Franz law, described as *K*_*e*_ *= LσT*, where *L* is the Lorenz number. As shown in Figure 4B, the Lorenz number was estimated from the empirical relation *L* = 1.5 + exp(–|*S*|/116).^38^ Figure 4C shows that, in general, *K*_*e*_ in the CPS, HP, and HPS samples initially decreased with temperature, and then increased. The observed exception was related to the CP sample whose *K*_*e*_ exhibited a near increasing trend throughout the measured range, while remaining significantly lower than the other samples due to its lower *σ*. In the HP and HPS samples, the oxidation-induced interfacial barriers suppressed electron transport, and resulted in a pronounced reduction in *K*_*e*_. As shown in Figure 4D, in general, *K*_*lat*_+*K*_*b*_ generally decreased with temperature and reached a minimum before increasing again. This behavior is attributed to enhanced phonon-phonon scattering near room temperature, which suppresses *K*_*lat*_.^39^ In addition, native point defects (antisites and vacancies) contribute to lattice thermal resistance.^40^ Such defects introduce mass fluctuation and local lattice distortion, thereby enhancing high-frequency phonon scattering and further suppressing *K*_*lat*_.^41^ At higher temperatures, Umklapp scattering approaches its limit, and the thermal excitation of charge carriers intensifies electron-phonon interactions, leading to a subsequent increase in *K*_*lat*_. In addition, *K*_*lat*_+*K*_*b*_ of the HPS sample remained lower than that of the CPS sample. This may be due to the dislocations introduced during HP, which effectively scatter mid- and high-frequency phonons, thereby reducing *K*_*lat*_,^10^ as well as a slight reduction in *K*_*lat*_ that was caused by interfacial barriers.

**Figure 5:**
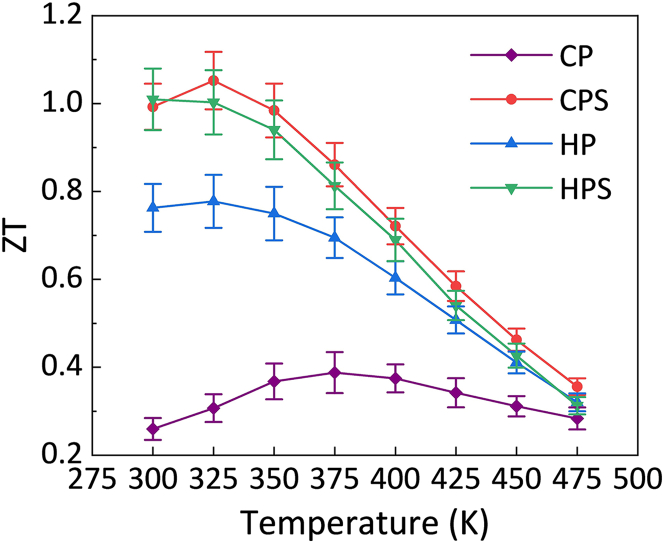
Temperature dependence of the figure of merit (ZT) in all Bi_0.3_Sb_1.7_Te_3_ samples prepared by different consolidation methods. Data are represented as mean ± SEM

Figure 5 shows the temperature-dependent *ZT* values for the BST samples fabricated via different consolidation methods. Among them, the CPS sample exhibited the highest *ZT* across the measured temperature range, benefiting from the simultaneously obtained high *PF* and relatively low *K*_*tot*_. It reached a maximum value of 1.05 at 325 K and then gradually decreased at higher temperatures. Within the 300–350 K range, the CPS sample maintained *ZT* values around unity, which were approximately 2–4 times greater than those of the CP sample. The HP and HPS samples achieved peak *ZT* values of 0.78 and 1.0 at 325 K and 300 K, respectively. Using an integration-based method, the average *ZT* values of the CP, CPS, HP, and HPS samples are 0.33, 0.99, 0.75, and 0.95, respectively. These results indicate that CP followed by sintering was the most effective consolidation route for enhancing the TE performance of BST powders in this study.

From a processing standpoint, CPS also offers practical advantages. In contrast to HPS, it avoids simultaneous high-temperature and pressing, thereby reducing energy consumption and equipment requirements. The hot-pressed series (HP/HPS) exhibited more pronounced oxidation-related features, suggesting that stricter atmosphere control and thermal management are necessary during high-temperature pressing. In comparison, CPS employed a simpler thermal profile while achieving ZT values comparable to HPS within experimental uncertainty. Furthermore, the separation of pressing and sintering steps allows independent optimization of powder processing and thermal treatment parameters, which is beneficial for scalable and reproducible manufacturing.

### Effect of particle size refinement on Bi_0.3_Sb_1.7_Te_3_ properties

#### Microstructural and chemical characterization

**Figure 6:**
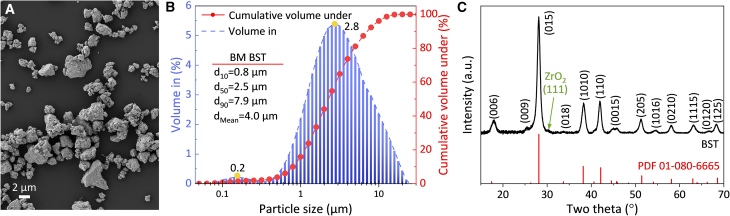
Characterization of ball-milled BST powder (A) SEM image. Scale bars represent 2 μm. (B) Particle size distribution. (C) XRD pattern.

To explore the influence of particle size on TE performance, the ground BST powder was further processed by ball milling, as described in the “materials and methods” section. The morphology and particle size distribution of the ball-milled BST powders are shown in Figures 6A and 6B, respectively. The characteristic cumulative size parameters, d_10_, d_50_, and d_90_, were 0.8, 2.5, and 7.9 μm, respectively. The milled particles remained irregular in shape, but their size distribution became significantly narrower and more uniform compared to the ground powder (shown in Figure 1). Figure 6C presents the XRD pattern of the milled powder, which agrees well with the reference data for Bi_0.3_Sb_1.7_Te_3_ (PDF# 01-080-6665). A minor peak near 2θ ≈ 30° was observed on the shoulder of the BST (015) peak in the milled powders. It can be attributed to ZrO_2_ (111) trace contamination, likely introduced by the milling balls and/or jar.

**Figure 7:**
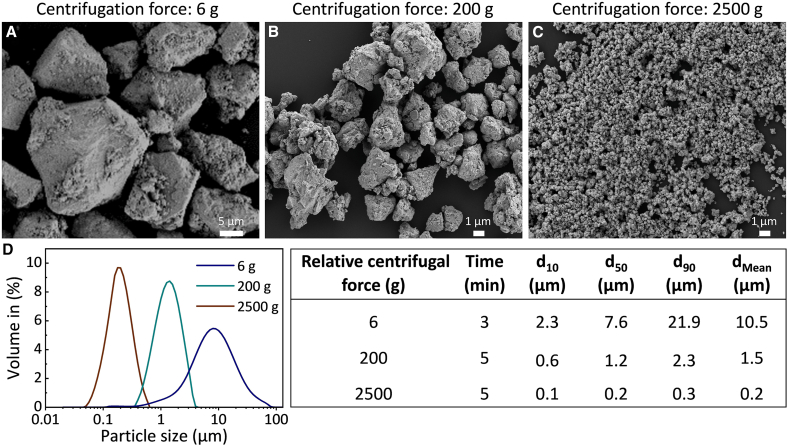
Centrifuged BST particles characterization (A–C) SEM images of centrifuged BST particles at relative centrifugal forces of (A) 6 g. Scale bars represent 5 μm. (B) 200 g. Scale bars represent 1 μm. (C) 2,500 g. Scale bars represent 1 μm. (D) Particle size distribution of the BST powder after centrifugation.

The milled BST powders were subsequently dispersed in IPA, followed by sonication and size-based separation via differential centrifugation at forces of 6, 200, and 2,500 g. The resulting powders exhibited mean (D[4,3]) particle sizes of approximately 10.5 μm, 1.5 μm, and 200 nm, respectively. Figure 7 illustrates the effect of centrifugation conditions on the particle size distribution and morphology of BST powders.

The centrifuged powders were compressed into bulk samples denoted CPS-10, CPS-1.5, and CPS-0.2, corresponding to their respective average particle sizes of 10.5, 1.5, and 0.2 μm. A summary of their microstructural features is provided in Table 1, together with data for the CPS sample derived from the ground powder. The CPS sample exhibited a broad and heterogeneous particle size distribution, reflecting its mixed-particle size structure. On the other hand, the application of ball milling followed by size-selective centrifugation enabled the production of more uniform microstructures in the CPS-10, CPS-1.5, and CPS-0.2 samples. As the particle size decreased, the corresponding CPS compacts exhibited lower densification, which is expected to influence both electron and phonon scattering, as will be discussed in section “thermoelectric characterization.”

**Table 1:** Comparing the microstructural features of cold-pressed and sintered Bi_0.3_Sb_1.7_Te_3_ samples prepared from powders with different particle size distribution characteristics

Sample name	CPS	CPS-10	CPS-1.5	CPS-0.2
Powder type	Manually ground	Milled and centrifuged	Milled and centrifuged	Milled and centrifuged
Average particle size	11.8 μm	10.5 μm	1.5 μm	0.2 μm
Relative density (%)	93.5	84.2	72.3	68.6
*F* factor	0.05	0.01	0.01	0.01
Microstructural schematic	–			

To assess grain orientation, XRD analysis was carried out on the consolidated samples. The resulting diffraction patterns (Data S5) were used to calculate the Lotgering orientation factor (*F*) using (Equation 3), (Equation 4), (Equation 5),

(Equation 3) $$F=\frac{P-P_{0}}{1-P_{0}}$$

(Equation 4) $$P=\frac{\sum I\left(hkl\right)}{\sum I_{total}}$$

(Equation 5) $$P_{0}=\frac{\sum I_{0}\left(hkl\right)}{\sum I_{total,0}}$$

where *P* and *P*_0_ denote the ratios of the summed intensities of the target (*00l*) plane to the total diffraction intensity for the bulk samples and corresponding powders, respectively. An *F* value of 0 indicates a fully random orientation, while a value of 1 represents perfect alignment along the (*00L*) plane. As listed in Table 1, the CPS sample prepared from ground powder exhibited a relatively low *F* value, suggesting that the particles (and grains) were randomly distributed without preferential orientation. Even lower *F* values were obtained in the CPS-10, CPS-1.5, and CPS-0.2 samples, confirming that grain orientation in the milled/centrifuged samples became more random and the microstructural isotropy was further improved. This isotropic behavior was validated through electrical property measurements performed in two orthogonal directions (parallel and perpendicular to the pressing axis). The negligible directional differences in the measured properties (Methods S1) provided strong evidence of structural isotropy in the milled/centrifuged samples.

**Figure 8:**
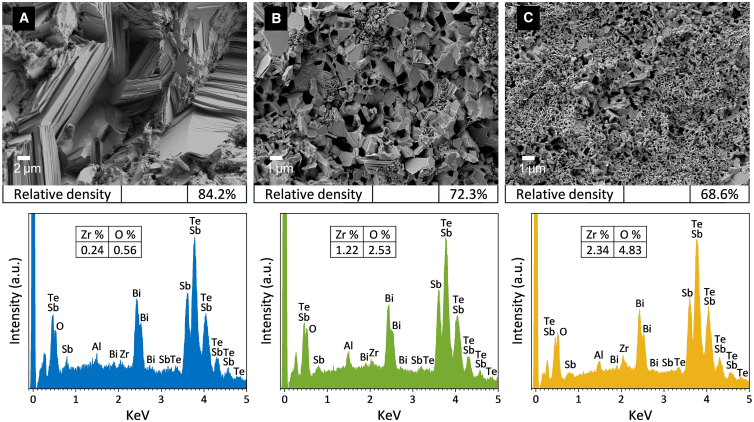
Cross-sectional SEM images and EDS spectra of particle size refined BST samples (A) CPS-10. Scale bars represent 2 μm. (B) CPS-1.5. Scale bars represent 1 μm. (C) CPS-0.2. Scale bars represent 1 μm.

Figures 8A–8C present cross-sectional SEM micrographs alongside the corresponding EDS spectra for the CPS-10, CPS-1.5, and CPS-0.2 samples. The SEM images indicate that the particle sizes in the consolidated samples closely match those of the starting powders, suggesting that the particles largely retained their initial size during the compaction and sintering stages. EDS analysis confirmed the presence of Bi, Te, and Sb in all samples, while the detected C, O, and Al peaks were attributed to the carbon tape, substrate, and SEM sample stage. Additionally, weak Zr signals were detected in the CPS-1.5 and CPS-0.2 samples. The corresponding quantitative analysis (atomic concentration) is provided in the inset tables of Figure 8. Trace zirconia (ZrO_2_) contamination is due to the high-energy ball milling and longer milling durations used for producing the finer powders, as discussed in Data S6.

Compared to the CPS-10 sample, which contained a small number of larger intergranular voids, the CPS-1.5 and CPS-0.2 samples displayed numerous and more uniformly distributed micro-/nano-scale pores and defects, consistent with their smaller particle sizes and lower relative densities. In general, smaller powder particles tend to form compacts with lower green densities under identical pressing conditions because higher surface energy and friction between particles hinder efficient rearrangement and packing. As a result, a broad size distribution favors more efficient packing, because the smaller particles fit in between the larger ones. Such defect-rich microstructures are expected to enhance phonon scattering and thus reduce *K*_*lat*_, which can be beneficial for TE performance.

**Figure 9:**
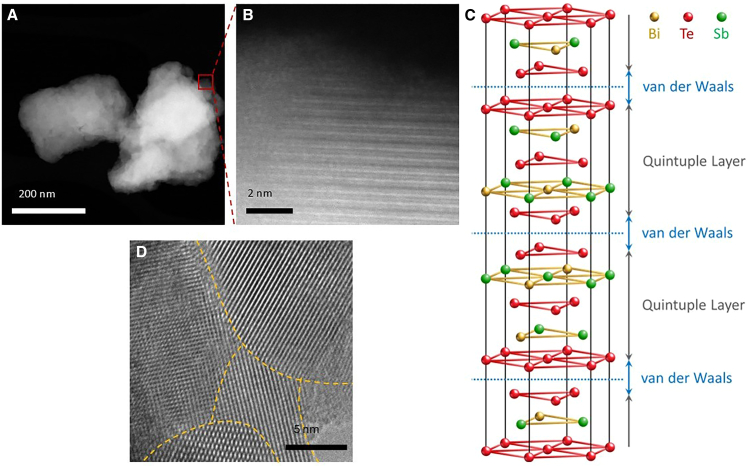
TEM characterization in CPS-0.2 sample (A) Dark field STEM image of CPS-0.2 sample. Scale bars represent 200 nm. (B) High magnification HAADF-STEM image confirming crystallinity. Scale bars represent 2 nm. (C) A schematic of the BST crystal lattice and its layered structure. (D) High-resolution TEM image of the CPS-0.2 sample, showing ordered grains and excellent crystallinity. Scale bars represent 5 nm.

To further examine the microstructural features, transmission electron microscopy (TEM) and scanning transmission electron microscopy (STEM) were employed to investigate grain boundaries and structural imperfections. Figure 9A shows a high-angle annular dark-field (HAADF) STEM image of the CPS-0.2 sample, where no obvious secondary phases were detected. A higher magnification image in Figure 9B displays well-defined lattice fringes, crystallinity, and the characteristic quintuple-layer stacking sequence of the material, consisting of Te-Bi/Sb-Te-Bi/Sb-Te layers, as schematically illustrated in Figure 9C. The sharp and continuous lattice fringes indicate minimal structural distortion and the retention of long-range atomic order. In addition, the representative high-resolution TEM image (Figure 9D) further validates the crystallinity over larger regions and clearly delineates grain boundary areas. As will be discussed in the next section (thermoelectric characterization), such boundaries are expected to act as effective phonon scattering centers, contributing to the reduction of *K*_*tot*_.

#### Thermoelectric characterization

**Figure 10:**
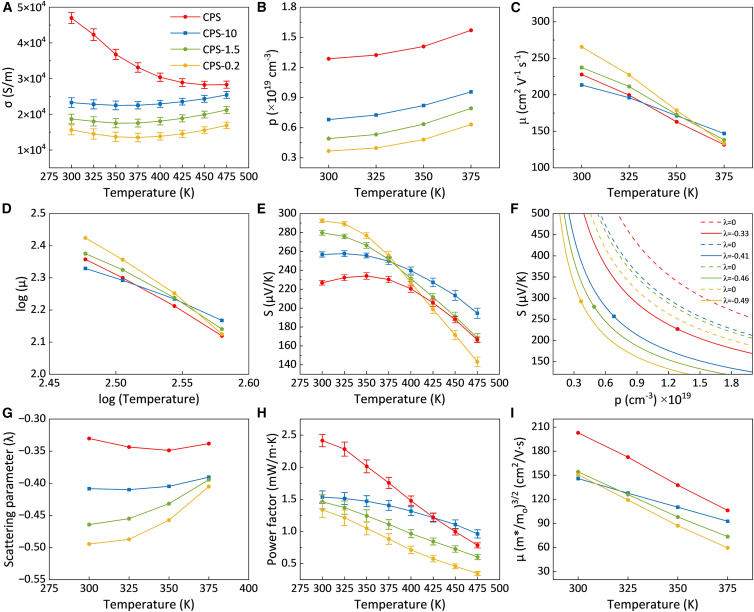
Electrical transport properties in cold-pressed and sintered samples with different particle sizes Data are represented as mean ± SEM. (A–C) Temperature dependence of (A) electrical conductivity, (B) charge carrier concentration, and (C) charge carrier mobility. (D) Measurement of parameter *x* in the temperature dependence of mobility described as *μ* ∝ *Tx*. (E) Temperature dependence of the Seebeck coefficient. (F) Seebeck coefficient versus charge carrier concentration (Pisarenko lines) at 300 K. (G–I) Temperature dependence of (G) SPB scattering parameter, (H) power factor, and (I) weighted mobility.

Figure 10 presents the temperature-dependent electrical transport properties of CPS samples with different particle sizes. As shown in Figure 10A, the *σ* had an inverse correlation with particle size. The *σ* decreased from 47 × 10^3^ S m^−1^ in the CPS sample to 15 × 10^3^ S m^−1^ in the CPS-0.2 sample at 300 K, corresponding to a reduction of over 68% (more details in Data S1). As shown in Figure 10B, Hall effect measurements revealed a substantial decrease in Hall carrier concentration with decreasing particle size. While enhanced carrier scattering primarily reduces *μ*, the reduction in *p* can be attributed to grain-boundary/interface effects. In fine-grained polycrystalline semiconductors, boundaries between two grains or particles can contain electrically active states that trap majority carriers and form depletion regions and potential barriers, leading to barrier-limited transport and an increased Hall coefficient.^42^^,^^43^^,^^44^^,^^45^ That reduces the Hall-effective carrier concentration extracted from *p*_H_ = 1/(eR_H_). Relative to the CPS sample, the *p* in CPS-10, CPS-1.5, and CPS-0.2 samples decreased by 47.1%, 61.8%, and 71.4%, respectively. A slight improvement in *μ* was observed in the smaller-particle samples (Figure 10C), but the large decrease in *p* was the dominant factor leading to the overall reduction in *σ*. This behavior stemmed from the lower relative densities and higher number of interface boundaries in the centrifuged samples, intensifying charge carrier scattering. Moreover, the *μ* of CPS-0.2 declined more rapidly with increasing temperature, indicating the synergistic effect of interface boundaries on phonon scattering at elevated temperatures. In contrast to the CPS sample, the centrifuged samples demonstrated a gradual rise in *σ* with temperature, especially at higher temperatures. This trend can be attributed to thermally activated hopping conduction across interface boundaries, as described by Equation 6:

(Equation 6) $$\sigma \left(T\right)∼T^{-\frac{1}{2}}e^{-\frac{E_{B}}{k_{B}T}}$$

in which, *E*_*B*_ is the interface boundary potential barrier. This positive temperature dependence becomes more evident in nanostructured samples due to the larger number of interface boundary barriers. However, as temperature rises, thermal activation facilitates carrier transport across these barriers, thereby diminishing the *σ* difference between samples with different particle sizes. For example, the *σ* of the CPS-0.2 sample at 300 K was ∼68% lower than that of the CPS sample, but the gap narrowed to ∼40% at 475 K.

The relationship between *μ* and temperature was previously described using Equation 1, with the slope of each line in Figure 10D corresponding to the exponent *x*. For the CPS sample, the slope was −2.32, while the slopes for the CPS-10, CPS-1.5, and CPS-0.2 samples ranged from −1.57 to −2.90. These values indicated that phonon scattering (likely involving a combination of acoustic and optical phonon interactions) was the dominant carrier scattering mechanism in all CPS-series samples.

Figure 10E shows how *S* varies with temperature for the different samples. The CPS sample exhibited a peak maximum at 350 K. As the particle size decreased, this peak shifted to lower temperatures, 325 K for CPS-10 and 300 K for both CPS-1.5 and CPS-0.2. This indicates that bipolar conduction became more prominent and set in at lower temperatures in the smaller-particle samples. This shift is consistent with the lower *p* and the stronger interface-boundary effects in these compacts. A clear trend of increasing *S* with decreasing particle size was observed at 300 K, which was primarily attributed to the reduction in *p*. In addition, the smaller-particle samples showed slightly higher mobilities at room temperature, reflecting the suppression of low-energy carriers by interfacial energy filtering at boundary barriers. This selective filtering reduced the effective *p* while allowing high-energy carriers to dominate, thus providing an additional enhancement of *S*. The CPS-0.2 sample reached the highest value of 292 μV K^−1^, followed by CPS-1.5 (279 μV K^−1^), CPS-10 (256 μV K^−1^), and CPS (226 μV K^−1^). Additionally, the presence of minor ZrO_2_ inclusions (introduced during ball milling) may have contributed to the formation of extra heterointerfaces, thus intensifying scattering and filtering mechanisms, particularly in the CPS-1.5 and CPS-0.2 samples. The similarity of the *S-T* curves for the CPS and CPS-10 samples was likely due to their comparable average particle sizes (11.8 and 10.5 μm, respectively). Under the Boltzmann transport approximation, this led to similar energy-dependent transport behavior. Differences in *S* values arose mainly from variations in particle size distribution and relative density of the samples, with CPS-10 exhibiting slightly more effective filtering of low-energy carriers and consequently a moderately higher *S*. Similarly, the apparent band gap *E*_*g*_ was estimated from the Goldsmid-Sharp relation. The CPS, CPS-10, CPS-1.5, and CPS-0.2 samples yielded band-gap values from 0.16 ± 0.1 eV. These results indicate that particle-size-refinement did not significantly modify the band structure of BST, and that the observed changes in transport behavior mainly originated from microstructural effects and defect-related scattering rather than from band-gap variation.

Figure 10F presents the Pisarenko plot at 300 K, constructed from Equation 2 for different scattering parameters. The SPB-Pisarenko analysis confirmed the inverse correlation between *S* and *p* (*S*∝*p*^−2/3^). The smaller-size particle compacts exhibited lower *p* and, therefore, higher *S*. All datapoints lay below the *λ* = 0 reference at their respective *p*, indicating a downward vertical shift of the Pisarenko line. Within the SPB framework, this can be attributed to a more negative *λ* and/or a smaller *m*∗, both of which diminish *S* at a given *p*. Such behavior is consistent with strong energy-dependent scattering arising from interface-boundary barriers. Figure 10G displays the calculated *λ* at higher temperatures. At 300 K, the smaller-particle samples showed more negative *λ* values, indicating a stronger energy dependence of carrier scattering (where low-energy carriers were preferentially scattered and high-energy carriers dominated the transport). This behavior is consistent with the energy filtering effect and helps to explain the enhanced *S*. With increasing temperature, *λ* shifted toward less negative values, suggesting a gradual weakening of the energy filtering effect. The effective barrier height also decreased and promoted phonon-dominated transport mechanisms. However, the simultaneous increase in *p*, accelerated degradation of *μ*, and the onset of bipolar conduction collectively dominated the transport behavior at higher temperatures, resulting in the observed decrease in both *S* and *σ*.

The effect of particle size on the temperature dependence of the *PF* is illustrated in Figure 10H. A general decreasing trend in *PF* was observed in all CPS samples, reflecting the inherent trade-off between *σ* and *S*. While the centrifuged samples exhibited enhanced *S*, the power factors of CPS-1.5 and CPS-0.2 samples remained lower than that of the ground CPS sample, as *σ* reduction was more pronounced than the gain in *S*. The CPS-10 sample had a smaller *PF* compared to the CPS sample up to 425 K due to its smaller *σ* values, but at higher temperatures the higher *S* partially compensated for the conductivity loss, and the difference in *PF* became less significant. At 300 K, the *PF* values of CPS-10, CPS-1.5, and CPS-0.2 were reduced by approximately 36%, 39%, and 44%, respectively, relative to the CPS sample. With increasing temperature, all samples exhibited a steady decline in the *PF*, but CPS-10 showed the slowest degradation, indicating the most favorable balance between enhanced *S* and reduced *σ*. By contrast, the CPS sample showed a rapid decline in the *PF* with temperature, driven mainly by its sharp *σ* decrease.

Figure 10I shows the temperature dependence of the *μ*_*w*_ for all samples in the range 300–375 K. Within the SPB model, the *PF* is related to *μ*_*w*_, as well as *p*, *m*∗, and *λ* through $PF\propto \mu_{w}{\left(1+\lambda \right)}^{2}{\left(m^{*}\right)}^{1/2}p^{-1/3}$. In this study, the ordering of *μ*_*w*_ for the centrifuged samples at room temperature did not fully coincide with that of the *PF*, mainly due to differences in *p* and *λ*. Therefore, while the *μ*_*w*_ generally reflects the transport characteristics of the carriers, the *PF* represented a multi-parameter coupled outcome and, in this context, the observed discrepancies between their trends are plausible.

**Figure 11:**
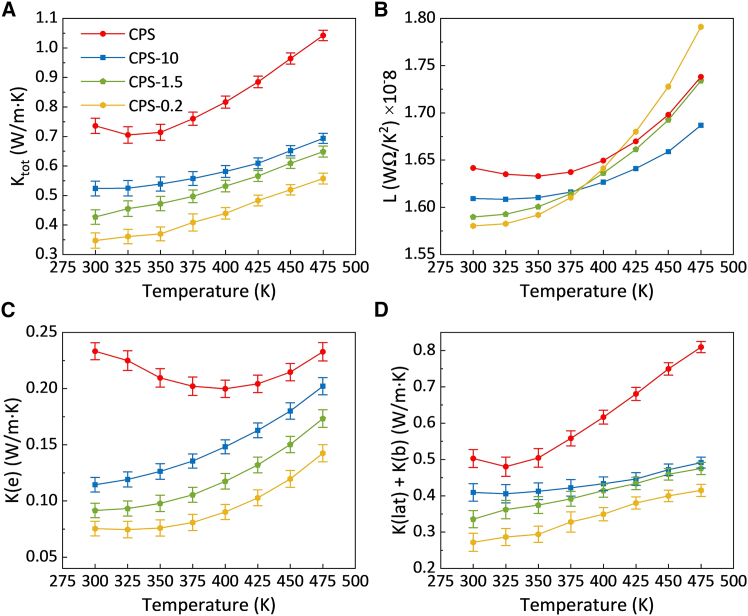
Thermal transport properties in cold-pressed and sintered samples with different particle sizes Data are represented as mean ± SEM. (A–D) Temperature dependence of (A) total thermal conductivity, (B) Lorentz number, (C) electronic component of thermal conductivity, and (D) lattice and bipolar components of thermal conductivity.

Figure 11 shows the temperature-dependent thermal transport properties in the CPS samples. As depicted in Figure 11A, the *K*_*tot*_ increased with temperature in all centrifuged samples. At 300 K, the *K*_*tot*_ values for CPS-10, CPS-1.5, and CPS-0.2 were reduced by approximately 29%, 42%, and 53%, respectively, compared to the CPS sample. This reduction is primarily attributed to the lower bulk densities and higher interface boundary density of the centrifuged samples, both of which enhance phonon and charge carrier scattering. The obtained *K*_*tot*_ values are at the lower end of those reported for p-type BST alloys, which typically range from 0.7 to 2.8 W m^−1^ K^−1^ at 300 K.^13^^,^^46^^,^^47^^,^^48^^,^^49^^,^^50^^,^^51^ In comparison, the CPS-series centrifuged samples (CPS-10, CPS-1.5, and CPS-0.2) exhibit exceptionally low thermal conductivity values of 0.52, 0.43, and 0.35 W m^−1^ K^−1^ at 300 K, respectively, highlighting the effectiveness of particle size engineering in suppressing heat transport. Figure 11B shows that with increasing temperature, *L* showed a slight upward trend in all centrifuged samples, especially for the smaller-particle compacts. This behavior deviates from the idealized prediction of the Wiedemann-Franz law and is likely influenced by inelastic charge carrier scattering and the presence of potential barriers at interfaces.

Figure 11C shows that *K*_*e*_ decreased with particle size refinement. At 300 K, the *K*_*e*_ values for CPS-10, CPS-1.5, and CPS-0.2 were 0.114, 0.092, and 0.076 W m^−1^ K^−1^, corresponding to reductions of 51%, 61%, and 67%, respectively, relative to the CPS value of 0.233 W m^−1^ K^−1^. This decline can be attributed to intensified charge carrier scattering at interface boundaries within the smaller-particle compacts.^52^ For all centrifuged samples, *K*_*e*_ increased with temperature, consistent with thermally activated carrier transport across interface boundary barriers. A comparable trend was observed for *K*_*lat*_*+K*_*b*_, as shown in Figure 11D, which also increased with temperature but decreased with particle size refinement. The overall reduction of *K*_*lat*_*+K*_*b*_ in centrifuged samples can be ascribed to extensive phonon scattering at interface boundaries and voids, effectively suppressing low- and mid-frequency phonons. Moreover, the minor ZrO_2_ phase could act as an additional phonon-scattering center due to acoustic impedance mismatch at the ZrO_2_/BST heterointerfaces, which is particularly effective in suppressing lattice thermal transport. Additionally, ball milling can introduce a large number of point defects (e.g., vacancies and antisite defects) in the crystal structure, which further contribute to high-frequency phonon scattering.^53^ It should be noted that *K*_*lat*_ below 0.25 W m^−1^ K^−1^ at 300 K has been achieved in BST alloys using advanced processing strategies (e.g., super-gravity),^54^ suggesting that further reductions in *K*_*lat*_ is feasible under suitable processing conditions. Nevertheless, the low *K*_*lat*_ obtained in this work using a scalable CPS-based route remains attractive from a practical manufacturing perspective. Taken together, these results indicate that at lower temperatures, electron scattering was more dominant, as evidenced by the greater suppression of *K*_*e*_. Conversely, at higher temperatures, phonon scattering became the dominant mechanism, as indicated by the stronger reduction in *K*_*lat*_ compared to *K*_*e*_, likely due to the increased contribution of phonon-phonon and defect-induced scattering processes under thermal excitation.

**Figure 12:**
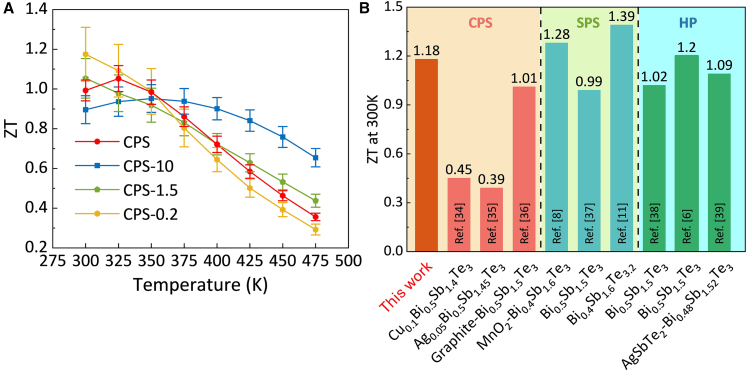
Thermoelectric performance of CPS-processed BST samples with different particle sizes and comparison with literature Data are represented as mean ± SEM. (A) Temperature dependence of the figure of merit (ZT) in cold-pressed and sintered samples with different particle sizes. (B) A comparison of ZT at 300 K for the CPS-0.2 sample in the present work with literature data on p-type bismuth telluride-based materials prepared by cold-press sintering, SPS, and HP.

Figure 12A shows the temperature dependence of *ZT* for the CPS series. At 300 K, the smallest-particle sample (CPS-0.2) delivered the highest TE performance (*ZT* ≈ 1.18), followed by CPS-1.5, CPS, and CPS-10. The superior room temperature *ZT* of CPS-0.2 originated from its reduced *K*_*tot*_ and enhanced *S*. However, excessive particle size refinement also introduced stronger carrier scattering and lowered the *p* at higher temperatures, which led to the *PF* decline and rapid *ZT* reduction. By contrast, CPS-10 achieved a more favorable trade-off as its *PF* decreased only slightly at higher temperatures while its *K* was substantially reduced. This balance enabled CPS-10 to maintain the most stable *ZT* in the 300–400 K range. The ground CPS sample initially benefited from high conductivity but suffered from a faster *PF* decay combined with a relatively higher *K*, which caused the *ZT* to decrease steeply upon heating. Beyond peak *ZT*, the average *ZT* were further evaluated in the practically relevant temperature range (300–375 K). The average *ZT* values for CPS, CPS-10, CPS-1.5, and CPS-0.2 were 0.99, 0.94, 0.95, and 1.02, respectively. This confirms that CPS-0.2 had the highest near-room-temperature efficiency. However, while CPS-0.2 was optimal for near room temperature applications, CPS-10 offered the best compromise between *PF* and *K*, rendering it more suitable for operation over a wider temperature window.

Figure 12B provides a comparison of the room temperature *ZT* of the CPS-0.2 sample with reported values for p-type bismuth telluride-based compounds and composites reported in the literature.^9^^,^^11^^,^^14^^,^^55^^,^^56^^,^^57^^,^^58^^,^^59^^,^^60^ The room temperature *ZT* of 1.18 achieved here exceeds previously reported values for cold-pressed and sintered samples, and is comparable to those fabricated by HP, even though it remains slightly lower than the best results reported for spark plasma sintered materials. This comparison highlights the effectiveness of the CPS strategy, which combines simplicity, lower energy consumption and cost, and scalability with competitive TE performance, making it a viable alternative to conventional high-temperature densification techniques.

In conclusion, this study systematically investigated the effects of consolidation technique and particle size on the microstructure and thermoelectric performance of p-type Bi_0.3_Sb_1.7_Te_3_. Manually ground Bi_0.3_Sb_1.7_Te_3_ powders were consolidated using four different methods: (1) CP, (2) cold pressing followed by vacuum sintering, (3) HP, and (4) hot pressing followed by vacuum sintering. Among these, the CPS samples exhibited the most favorable microstructural features (high relative density and smaller particle size after sintering) which resulted in higher electrical conductivity, enhanced Seebeck coefficient, and reduced thermal conductivity, leading to a peak *ZT* of ∼1.05 at 325 K. In contrast, the HP and HPS samples showed degraded transport performance due to slight oxidation at higher temperatures, which introduced additional carrier- and phonon-scattering barriers.

For further optimization, particle size refinement was achieved by combining ball milling with differential centrifugation, yielding powders with average particle sizes of ∼10, 1.5, and 0.2 μm. Bulk samples prepared via the optimized CPS method from these powders demonstrated clear improvements in electrothermal transport: interfacial energy filtering of low-energy carriers enhanced the Seebeck coefficient, while interface-boundary scattering effectively suppressed thermal conductivity. Among these, the CPS-0.2 sample with an average particle size of ∼0.2 μm achieved the best performance, reaching a high *ZT* of 1.18 at 300 K.

Finally, cold-press sintering was demonstrated to be an efficient and energy-saving consolidation method that preserved the intrinsic particle features. In combination with microstructural engineering via particle-size refinement, this approach enabled simultaneous optimization of electrical and thermal transport, and led to a significant enhancement of the near-room-temperature thermoelectric performance.

## Outlook

Looking forward, the process-structure-property framework established in this work can be extended to other TE material systems, providing a scalable route for microstructural optimization beyond p-type Bi_0.3_Sb_1.7_Te_3_. When combined with established strategies such as carrier concentration tuning, mobility engineering, and defect modulation, further enhancement of TE performance should be achievable. From a device perspective, the optimized CPS route enables scalable fabrication of TE legs and supports module-level optimization, including systematic tuning of leg geometry and inter-leg spacing to improve power output. For flexible TEGs, optimization of filler and encapsulation layers to better manage heat flow and mechanical deformation represents a promising direction. Ultimately, integrating such flexible devices into wearable platforms, such as smart textiles, could enable continuous harvesting of body heat to power low-energy electronics.

### Limitations of the study

Despite the demonstrated effectiveness of the CPS route in optimizing the microstructure and TE performance of p-type Bi_0.3_Sb_1.7_Te_3_, certain limitations should be acknowledged. First, the present study focuses exclusively on a single material composition within the Bi-Sb-Te system. Although the proposed process-structure-property framework provides valuable insight into microstructural engineering, its transferability to other TE material systems (e.g., non-Bi-Te-based compounds) should be experimentally validated. Differences in phase stability, defect chemistry, and interface/grain boundary interactions with charge carriers may lead to distinct structure-property relationships that require independent optimization. Second, the performance improvements reported here primarily arise from microstructural control via CPS. Other well-established strategies, such as carrier concentration tuning, band structure engineering, mobility enhancement, and defect modulation, were not systematically integrated. As a result, the achievable TE performance may not yet represent the intrinsic upper limit of the material system.

## Resource availability

### Lead contact

Requests for further information and resources should be directed to and will be fulfilled by the lead contact, Amir Pakdel (pakdela@tcd.ie).

### Materials availability

This study did not generate new unique reagents.

### Data and code availability

• All data reported in this paper will be shared by the lead contact upon request.
• This study did not generate new codes.
• Any other items related to this study will be shared by the lead contact upon request.

## Acknowledgments

R.X. acknowledges the China Scholarship Council (CSC) for his PhD scholarship. A.P. would like to acknowledge the financial support of 10.13039/501100001602Science Foundation Ireland (SFI), grant no. 18/SIRG/5621. The authors would like to thank Robert Dunbar and Gerry Byrne (TCD School of Engineering), Fabien Veillon (10.13039/100013349Laboratoire CRISMAT), and the Advanced Microscopy Laboratory (TCD) for their technical support. This work was supported by the 10.13039/501100001602Science Foundation Ireland (SFI), grant no. 18/SIRG/5621.

## Author contributions

Conceptualization, R.X. and A.P.; methodology, R.X. and A.R.; formal analysis, R.X., A.R., F.G., and J.L.; data curation, R.X.; writing, R.X. and A.P.; supervision, A.P.; project administration, A.P.; funding acquisition, A.P.

## Declaration of interests

The authors declare no conflict of interest. The funders had no role in the design of the study; in the collection, analyses, or interpretation of data; in the writing of the manuscript, or in the decision to publish the results.

## STAR★Methods

### Key resources table

**Table:** 

REAGENT or RESOURCE	SOURCE	IDENTIFIER
**Chemicals, peptides, and recombinant proteins**		

p-type Bi_0_._3_Sb_1_._7_Te_3_ ingot	Thermonamic Electronics	TIG-BiTe-P/*N*-1
2-Propanol	sigmaaldrich	67-63-0
hydrochloric acid	Honeywell	7647-01-0

**Software and algorithms**		

Origin	OriginLab	Origin 2023

**Other**		

Planetary ball mill	Retsch GmbH	PM-100
Tip sonication	FIsherbrand	FB120
Centrifuge	Eppendorf	5804
Uniaxial testing machine	Instron	5589
Furnace	Nabertherm GmbH	Tube Furnace
Laser diffraction particle size analyzer	Malvern Instruments Ltd.	Mastersizer 2000
X-ray diffractometer	Bruker	D8 Advance
Scanning electron microscopy	Zeiss	Ultra Plus
Transmission electron microscope	FEI Titan	80-300 kV FEG S/TEM
Linseis LSR-3 system	Linseis	Linseis LSR-3
Laser flash apparatus	NETZSCH	LFA 457
Differential scanning calorimeter	Perkin Elmer	DSC 8000

### Experimental model and study participant details

No experimental models or study participants were used in the study.

### Method details

#### Powder preparation

The starting material was a p-type Bi_0.3_Sb_1.7_Te_3_ ingot, sourced from Thermonamic Electronics (Jiangxi Corp., Ltd., Nanchang, China). The ingot was initially cut into smaller pieces and manually ground using a mortar and pestle for 6 h to produce granular powder (more details in Methods S2).

The subsequent ball milling was carried out using a planetary ball mill (Retsch GmbH, PM-100, Germany). The ground BST powder was loaded into a 50 mL zirconia jar with zirconia milling balls. Milling was performed for 10 h at 600 rpm with a ball-to-powder weight ratio of 20:1. To aid particle fragmentation and minimize agglomeration or caking due to swelling, 20 μL of IPA was added to the jar. The milling process included 30-min milling cycles followed by 10-min pauses, with regular rotation reversals, to maintain the powder temperature below 323 K.

The ball-milled BST powders were dispersed in IPA at a concentration of 1.5 g/50 mL and subjected to 20 min of tip sonication. The resulting suspension was centrifuged at different relative centrifugal forces of 6 g for 3 min, 200 g for 5 min, and 2500 g for 5 min. Powders with different particle-size distributions were obtained by collecting the precipitates at each stage. These powders were subsequently washed with a 5 vol % HCl solution, rinsed with deionized water and IPA, and dried at (323 K) for 12 h under a nitrogen atmosphere to minimize oxidation. The average particle sizes of the fractions obtained at 6 g, 200 g, and 2500 g batches were 10.5 μm, 1.5 μm, and 0.2 μm, respectively.

#### Powder compaction and sintering

The manually-ground BST powders were compacted into bulk samples using a uniaxial testing machine (Instron 5589, USA) equipped with a heating system for temperature control. A thermocouple inserted into a small hole in the die was used for real-time monitoring, and graphite sheets were placed between the die and plunger before each compression. Both CP and HP procedures utilized 5 g of BST powder in a 10 × 10 mm stainless-steel square die. CP was performed under 750 MPa for 30 min at room temperature, while HP involved heating the die to 693 K in ambient air and atmospheric conditions, applying 80 MPa for 15 min, and allowing the system to cool naturally. The resulting pellets were labeled as CP and HP, respectively. Following the pressing process, selected CP and HP samples were further sintered under vacuum at 693 K for 5 h without pressure (parameter optimization detailed in Data S7), resulting in samples designated as CPS and HPS, respectively. The ball-milled powders were compressed into bulk samples via CPS, producing samples denoted CPS-10, CPS-1.5, and CPS-0.2, corresponding to average particle sizes of 10.5 μm, 1.5 μm, and 0.2 μm, respectively.

#### Materials characterization

The particle size and distribution analysis of both manually-ground and ball-milled BST powders was conducted using a laser diffraction particle size analyzer (Mastersizer 2000, Malvern Instruments Ltd., UK). Phase identification was carried out using a powder X-ray diffractometer (Bruker D8 Advance) equipped with Cu Kα radiation. Morphology and chemical composition of BST powders and cross-sectional surfaces of bulk samples were analyzed via a scanning electron microscopy (SEM, Zeiss Ultra Plus, Germany) operating at 20 kV, coupled with a Bruker XFlash7100 energy-dispersive X-ray spectrometer (EDS). Structural analysis was performed using a high-resolution field emission transmission electron microscope (FEI Titan 80–300 kV FEG S/TEM) operated at 300 kV with an annular STEM detector.

The electrical resistivity and Seebeck coefficient were simultaneously measured using a Linseis LSR-3 system under a helium atmosphere over the temperature range 300–475 K. The thermal diffusivity (*D*) was measured using a laser flash apparatus (LFA 457), while specific heat capacity (*C*_*p*_) was determined with a differential scanning calorimeter (PerkinElmer DSC 8000). The density (*ρ*) of the bulk samples was evaluated using the Archimedes’ method, and thermal conductivity (*K*) was calculated from the relation*: K = D ρ C*_*p*_.

### Quantification and statistical analysis

No statistical analysis was used in the study.
