# Supplementary material for: Cold-press sintering and particle-size engineering of Bi0.3Sb1.7Te3 for enhanced thermoelectric performance
Source: iScience. 2026 Mar 31;29(5):115544. doi: 10.1016/j.isci.2026.115544 (PMC13091770; doi:10.1016/j.isci.2026.115544)
Supplement: Document S1. Data S1–S7 and Methods S1–S2 [file mmc1.pdf]

iScience, Volume *29*

Supplemental information

Cold-press sintering and particle-size engineering of $Bi_{0.3}Sb_{1.7}Te_3$ for enhanced thermoelectric performance

Ruifeng Xiong, Jeremy Lemoigne, Aran Rafferty, Franck Gascoin, and Amir Pakdel

Supplementary information

Table of Contents

Data S1. Relative density and thermoelectric properties

Data S2. EDS elemental mapping of HP sample

Data S3. Band gap calculations

Data S4. Pisarenko lines analysis

Data S5. XRD patterns of CPS samples

Methods S1. Isotropic thermoelectric properties of CPS samples

Data S6. EDS elemental mapping of CPS-0.2 sample

Methods S2. Sample preparation

Data S7. Optimization of sintering temperature and time

Supplementary references

Data S1. Relative density and thermoelectric properties of all samples at 300 K

As shown in Table S1, the consolidation route and particle-size distribution have a clear influence on the room-temperature physical and thermoelectric properties of the $Bi_{0.3}Sb_{1.7}Te_3$ samples. To facilitate direct comparison across different consolidation routes and particle-size distributions, the relative density, *σ*, *S*, *K*, *PF*, and *ZT* at 300 K are listed.

The CPS sample exhibited the highest electrical conductivity and a relatively high density, reflecting the combined effects of cold pressing and subsequent sintering on electronic transport. The CPS and HPS samples exhibited comparable *ZT* values at room temperature, yet with increasing temperature the CPS sample eventually surpassed the HPS sample in *ZT*. In contrast, the CPS series (CPS, CPS-10, CPS-1.5, CPS-0.2) showed progressively reduced density, which is consistent with their lower electrical conductivities but higher Seebeck coefficients. Thermal conductivity generally decreased with lower density, contributing to the improved *ZT* values in the CPS-1.5 and CPS-0.2 samples.

Table S1. Summary of the relative density and thermoelectric properties of $Bi_{0.3}Sb_{1.7}Te_3$ samples prepared from powders with different consolidation routes and particle size distribution characteristics.

Sample	Relative density (%)	*σ* ($S\ m^{-1}$)	*S* ($\mu V\ K^{-1}$)	*PF* ($mW\ m^{-1}\ K^{-1}$)	*K* ($W\ m^{-1}\ K^{-1}$)	*ZT*
CP	87.6	8838.1	220.3	0.43	0.50	0.26
CPS	93.5	46963.1	226.8	2.41	0.74	1.0
HP	90.2	25735.1	248.8	1.59	0.64	0.76
HPS	91.7	33224.6	239.8	1.91	0.57	1.0
CPS-10	84.2	23282.7	256.8	1.54	0.52	0.89
CPS-1.5	72.3	18690.9	279.6	1.46	0.43	1.05
CPS-0.2	68.6	15635.7	292.5	1.34	0.35	1.18

Figure S1 illustrates the systematic dependence of relative density, electrical transport properties, thermal transport behavior, and the resulting TE performance of the CPS series as a function of average particle size. As shown in Figure S1(a), the relative density decreases monotonically with decreasing particle size, indicating reduced packing efficiency and increased porosity in finer-particle samples. This density reduction plays a critical role in governing both electrical and thermal transport.

Figure S1(b) shows the evolution of electrical properties with particle size. With decreasing particle size, accompanied by the reduction in relative density, the *σ* decreases significantly, while the *S* increases markedly. This opposite trend reflects enhanced carrier scattering at grain boundaries and interfaces introduced by particle size refinement. However, the decrease in *σ* is more pronounced than the increase in the *S*, resulting in an overall reduction of the *PF* with decreasing particle size.

The effect of particle size on thermal transport is presented in Figure S1(c). Both the *K* and the K_{lat}+K_b decrease systematically as the particle size is reduced. In particular, the pronounced suppression of K_{lat} highlights the effectiveness of particle-size-induced phonon scattering and interface effects, which constitute the central hypothesis of this work.

As a combined consequence of the competing trends in electrical and thermal transport, the dimensionless figure of merit *ZT* exhibits an overall increasing trend with decreasing particle size, as shown in Figure S1(d). Despite the deterioration in *σ*, the strong reduction in K_{lat} dominates the thermoelectric performance, contributing to the improved *ZT* values observed in the CPS-1.5 and CPS-0.2 samples. These results establish a clear and quantitative link between processing-induced particle size control, the resulting microstructural characteristics (density and interfaces), and the measured TE properties, thereby directly supporting the central thesis that phonon scattering

engineered through particle size and interfaces is an effective strategy for enhancing TE performance.

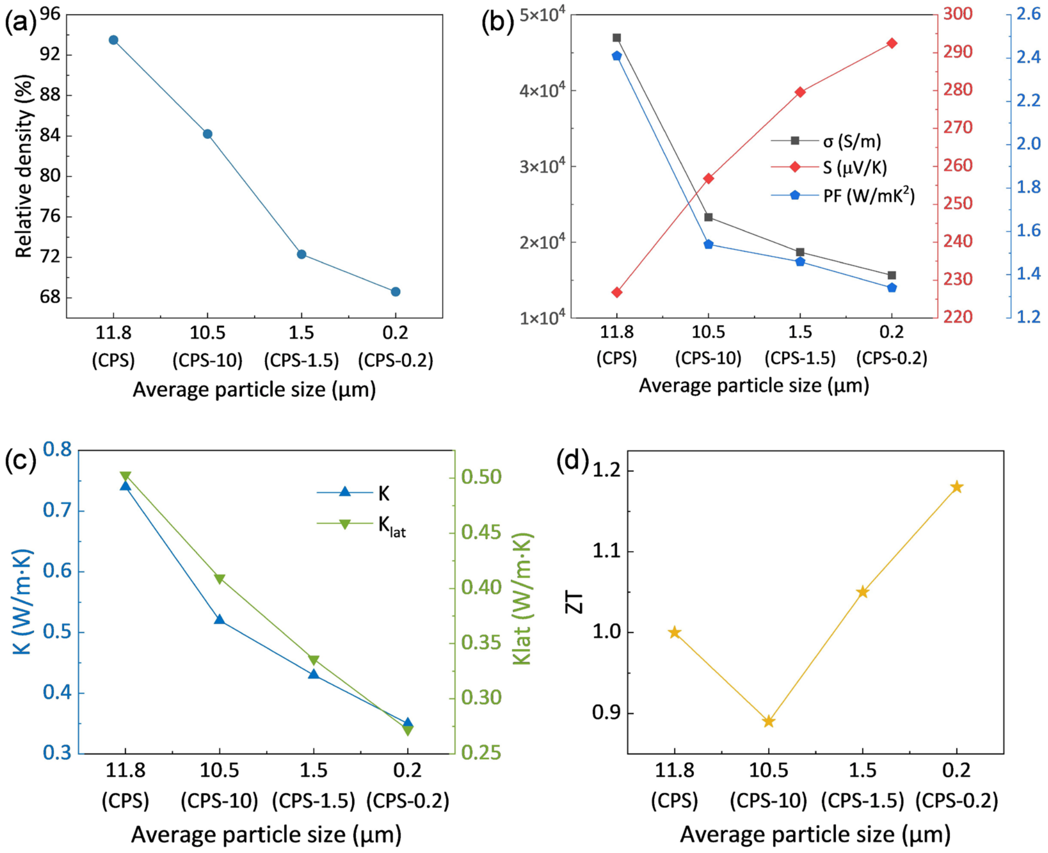

Figure S1. Average particle size dependence of (a) relative density, (b) electrical properties, (c) thermal conductivity and (d) thermoelectric figure of merit ZT of all CPS series samples at 300 K.

Data S2. EDS elemental mapping of HP sample

Figure S2 shows representative EDS elemental maps of the HP sample revealing a BST matrix decorated with many small particles. These O-rich regions spatially correlate with Sb-enriched zones, while showing reduced Bi and Te intensity. This is consistent with the formation of Sb-rich oxide phases (antimony oxides) at the surface of the BST grains during hot pressing. The oxide fraction is small and mainly confined to near-surface regions; therefore, it is not clearly resolved in the XRD patterns but is readily detected by EDS. This observation supports the interpretation of the stronger O peaks in the bulk EDS spectra of HP and HPS samples as arising from surface oxidation rather than from an oxygen-containing bulk phase.

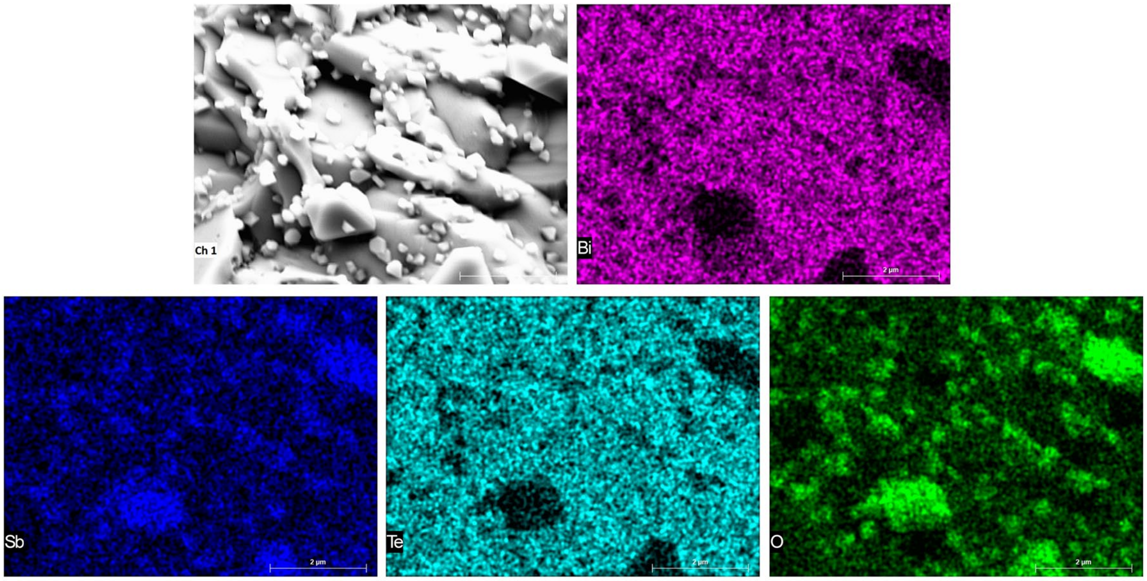

Figure S2. SEM image and corresponding EDS elemental maps of of Bi, Sb, Te, and O for the HP sample. The scale bar represents 2 μm.

Data S3. Estimation of the apparent band gap using the Goldsmid–Sharp relation

To further evaluate whether different consolidation routes and particle-size refinement steps modified the intrinsic electronic structure of $Bi_{0.3}Sb_{1.7}Te_3$, the apparent band gap (E_g) was extracted from the temperature dependence of *S* using the Goldsmid–Sharp relation: [1]

$$E_g \approx 2q|S|_{max}T_{max} \quad (1)$$

where q is the elementary electric charge, S_{max} is the maximum *S* obtained prior to the onset of bipolar conduction, and T_{max} is the temperature at which this maximum occurs. This method provides an estimation of the transport-relevant band gap in narrow-bandgap TE semiconductors, particularly those dominated by intrinsic excitation at high temperatures.

Table S2. Summary of S_{max} , T_{max}, and E_g values

Sample	S_{max} (μV K^{-1})	T_{max} (K)	E_g (eV)
CP	229.6	350	0.161
CPS	234.0	350	0.164
HP	250.3	325	0.163
HPS	243.5	325	0.158
CPS-10	257.6	325	0.1675
CPS-1.5	279.6	300	0.1678
CPS-0.2	292.5	300	0.1755

Table S2 shows the maximum *S* for each sample and the corresponding temperature at which it occurs, from which E_g can be calculated using the above relation. For all consolidation routes (CP, CPS, HP, HPS), E_g remains grouped between 0.158–0.164 eV, indicating that consolidation temperature, pressure differences, relative density, and microstructural variations (grain boundary density, porosity) do not significantly change the intrinsic band structure of p-type Bi–Sb–Te. Similarly, particle-size refinement series (CPS, CPS-10, CPS-1.5, and CPS-0.2), with average particle sizes from ~10 μm down to ~200 nm, also obtained E_g values with the range of 0.164–0.176 eV. The preserved band-gap values across both consolidation and size-engineering routes confirm that the observed differences in TE transport primarily arise from microstructural effects (grain size, porosity, grain-boundary density) and defect-related scattering, rather than from any modification of the intrinsic electronic band structure.

Data S4. Pisarenko lines analysis

The Seebeck coefficient was analyzed using the SPB model within the framework of Boltzmann transport theory, incorporating an energy-dependent carrier relaxation time, it can be expressed as:

$$S = \frac{k_B}{q}\left[\frac{(2+\lambda)F_{\lambda+1}(\eta)}{(1+\lambda)F_{\lambda}(\eta)} - \eta\right] \quad (2)$$

where λ is the scattering parameter that characterizes the energy dependence of carrier scattering and takes the value of 0 for acoustic phonon scattering, 1/2 for neutral impurity scattering, and 2 for ionized impurity scattering. $\eta = E_F/k_BT$ is the reduced Fermi level and $F_i(\eta)$is the Fermi integral of order i. This relation indicates that the Seebeck coefficient depends solely on the reduced chemical potential η and the scattering parameter λ.

By assuming dominant acoustic phonon scattering (λ = 0), the reduced Fermi level η was numerically extracted from the experimentally measured Seebeck coefficient at each temperature. The effective mass m^* was then determined using the corresponding carrier concentration obtained from Hall measurements, it can be expressed as:

$$m^* = \frac{h^2}{2k_BT}\left(\frac{p}{4\pi F_{1/2(\eta)}}\right)^{2/3} \quad (3)$$

Based on the extracted η and m^*, theoretical Pisarenko lines describing the relationship between the Seebeck coefficient and carrier concentration were constructed at different temperatures. Comparisons between experimental data and these Pisarenko lines allow assessment of the effective mass, dominant scattering mechanisms, and potential deviations from the SPB approximation.

Figure 3(f) in the manuscript shows the Pisarenko lines for samples at 300 K. The solid lines represent λ=0 (that is, charge scattering only by acoustic phonons) and the dashed lines show the experimental S and p data and the corresponding λ calculated from the following equation:

$$S = (1+\lambda)\frac{8\pi^2 k_B^2 T}{3eh^2} m^* \left(\frac{\pi}{3p}\right)^{2/3} \quad (4)$$

In this study, the experimentally measured carrier concentrations fall within a regime where a single band is expected to dominate electronic transport, justifying the applicability of the SPB model.

It should be noted that at higher carrier concentrations or elevated temperatures, deviations from the SPB approximation may arise due to multi-band contributions or band nonparabolicity, which are known to occur in Bi–Te–based systems. Nevertheless, within the carrier concentration and temperature ranges investigated here, no pronounced deviation from the Pisarenko relationship is observed, indicating that the SPB model remains an appropriate and physically meaningful approximation for interpreting the experimental Seebeck coefficient data.

To contextualize the SPB-based Pisarenko analysis, representative BST literature values of the m^* and scattering-related parameters were compiled, as shown in Table S3. In BST reports, SPB/Pisarenko fittings typically yield m^* values on the order of ~0.5–1.6 m_0, although the extracted values depend on composition, texture, and microstructure/defect engineering. Moreover, scattering-parameter analyses for p-type BST alloys have shown that the apparent scattering parameter can deviate from an idealized single mechanism (e.g., purely acoustic phonon scattering), reflecting mixed scattering contributions and/or additional interfacial potential barriers introduced by processing.

In the present work, the obtained parameters (m^* ≈ 0.69–1.08 m_0 and λ ≈ −0.31 to −0.49) are consistent with the above ranges. The gradual reduction of m^* from the coarse CPS sample to the finest-particle CPS-0.2 compact is reasonable considering that particle-size refinement increases interface density and defect/interface states, which can alter the energy-dependent transport and shift the Pisarenko trend without requiring a fundamental change in the intrinsic band structure (also consistent with the nearly unchanged Eg extracted from Goldsmid–Sharp analysis). Meanwhile, the

negative λ values can be understood as an "effective" descriptor capturing the enhanced energy dependence of carrier relaxation in the presence of abundant particle–particle interfaces and defect-associated scattering/trapping.

Table S3. Comparison of reported density-of-states effective mass and scattering-related parameters for p-type Bi–Sb–Te alloys based on SPB/Pisarenko analyses.

Material	Scattering Parameter	m_*/m_0	Ref.
$Bi_{0.3}Sb_{1.7}Te_3$	-0.31 to -0.49	0.69 to 1.08	This work
$Bi_{0.5}Sb_{1.7}Te_3$	—	0.52 to 0.65	[2]
$Bi_{0.4}Sb_{1.6}Te_3$	—	1.03	[3]
$Bi_{0.4}Sb_{1.6}Te_3$	—	1.2 to 1.6	[4]
$Bi_{0.5}Sb_{1.5}Te_3$	—	0.6 to 1.1	[5]
$Bi_{0.5}Sb_{1.5}Te_3$	—	1.1	[6]
$Bi_{0.42}Sb_{1.58}Te_3$	—	1.01 to 1.22	[7]
$Bi_{0.5}Sb_{1.5}Te_3$	—	1 to 1.2	[8]
$Bi_{0.5}Sb_{1.5}Te_3$	—	0.84 to 1.02	[9]
$Bi_{0.3}Sb_{1.7}Te_3$	—	1 to 1.58	[10]
$Bi_{0.5}Sb_{1.5}Te_3$	—	0.77 to 1.09	[11]
$Bi_{0.5}Sb_{1.5}Te_3$	—	0.68 to 1.25	[12]
$Bi_{0.5}Sb_{1.5}Te_3$	-0.17 to -0.34	—	[13]
$Bi_{0.5}Sb_{1.5}Te_3$	-0.32 to -0.5	1.12	[14]
$Bi_{0.3}Sb_{1.7}Te_3$	-0.36 to -0.52	1.04	[15]
$Bi_{0.3}Sb_{1.7}Te_3$	-0.6 to -1.06	—	[16]
$Bi_{0.5}Sb_{1.5}Te_3$	-0.9 to -2.2	—	[17]
Bi_2Te_3	-0.8 to 0.1	—	[18]

Data S5. XRD patterns of cold-press sintered samples

Figure S3 presents the XRD patterns of various CPS samples. In all cases, the diffraction peaks of the CPS samples show good agreement with the standard JCPDS card for $Bi_{0.3}Sb_{1.7}Te_3$. Based on the XRD results of the samples, the orientation factor F was calculated using Equations (3–5) provided in the manuscript.

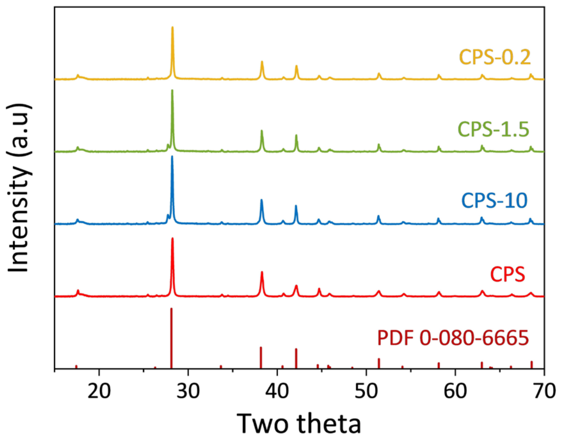

Figure S3. XRD patterns of the cold-press sintered samples.

Methods S1. Isotropic thermoelectric properties of CPS samples

Figure S4(a) shows typical sample preparation and measurement methodology. Cold-press sintering was used to manufacture a 10 × 10 × 10 mm cube sample using manually-ground BST powder, and its electrical properties were measured along two orthogonal directions. These directions refer to sample facets perpendicular and parallel to the pressing axis. Figure S4 (b-d) presents the temperature dependence of the electrical conductivity, Seebeck coefficient and power factor of the sample in the range of 300–475 K. The obtained results showed minor variations between the two directions, thus confirming the isotropy of the CPS sample. The maximum differences in electrical conductivity, Seebeck coefficient and power factor were 4.8%, 2.5% and 8.8%, respectively.

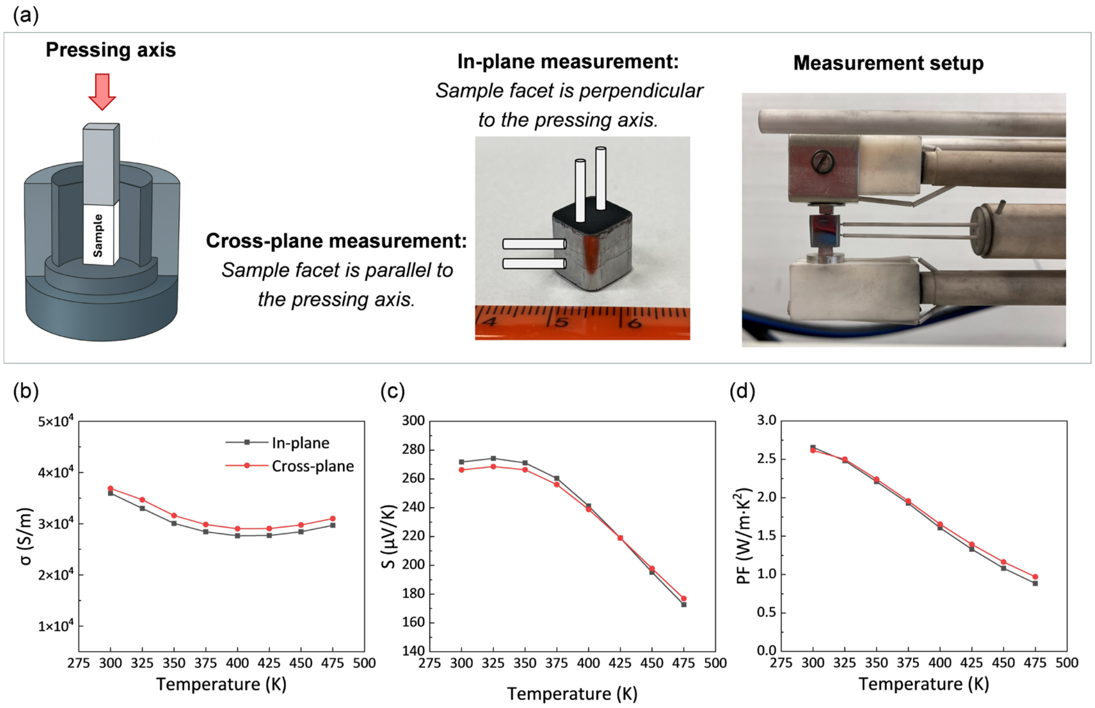

Figure S4. Sample preparation and measurement details for in-plane and cross-plane directions. Comparing the temperature dependence of (b) electrical conductivity, (c) the Seebeck coefficient and (d) power factor for in-plane and cross-plane directions.

Data S6. EDS elemental mapping of CPS-0.2 sample

Figure S5 shows representative EDS elemental maps of the CPS-0.2 sample confirming minor presence of zirconia (ZrO_2) after ball milling in a zirconia jar using zirconia milling balls.

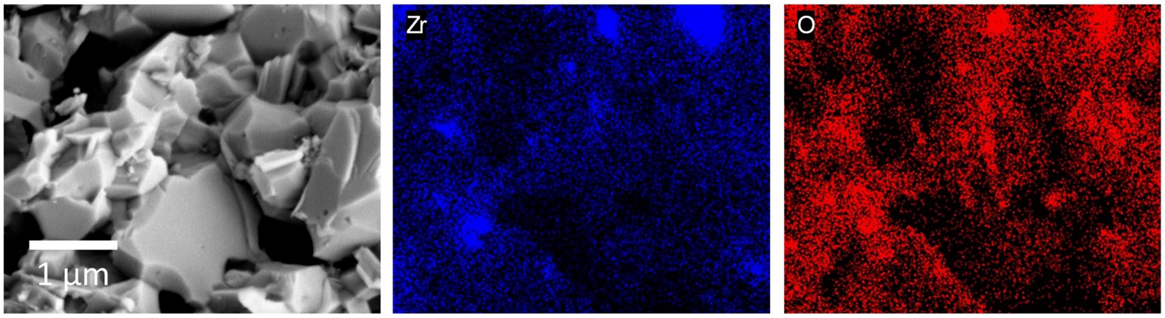

Figure S5. SEM image and corresponding EDS elemental mapping of CPS-0.2 sample. The scale bar represents 1 µm.

Methods S2. Sample preparation of $Bi_{0.3}Sb_{1.7}Te_3$ bulk samples

The preparation of $Bi_{0.3}Sb_{1.7}Te_3$ samples involved two processing routes, as illustrated in Figure S6. In the first route, bulk ingots of $Bi_{0.3}Sb_{1.7}Te_3$ were manually ground in an agate mortar to obtain coarse powders (Figure S6(a)), which were then directly consolidated via four different densification processes: cold pressing (CP), cold pressing followed by vacuum sintering (CPS), hot pressing (HP), and hot pressing followed by vacuum sintering (HPS) (Figure S6(c)). In the second route, the manually ground powders were further subjected to ball milling, during which the repeated impact and shear forces between grinding balls and powder particles promoted size reduction. The resulting suspension was subsequently centrifuged into three distinct fractions with average particle sizes of ~10.5 μm, ~1.5 μm, and ~200 nm (Figure S6(b)). The fractionated powders were then compacted using the CP method and vacuum sintered in a tubular furnace to fabricate $Bi_{0.3}Sb_{1.7}Te_3$ bulk samples (Figure S6(c)).

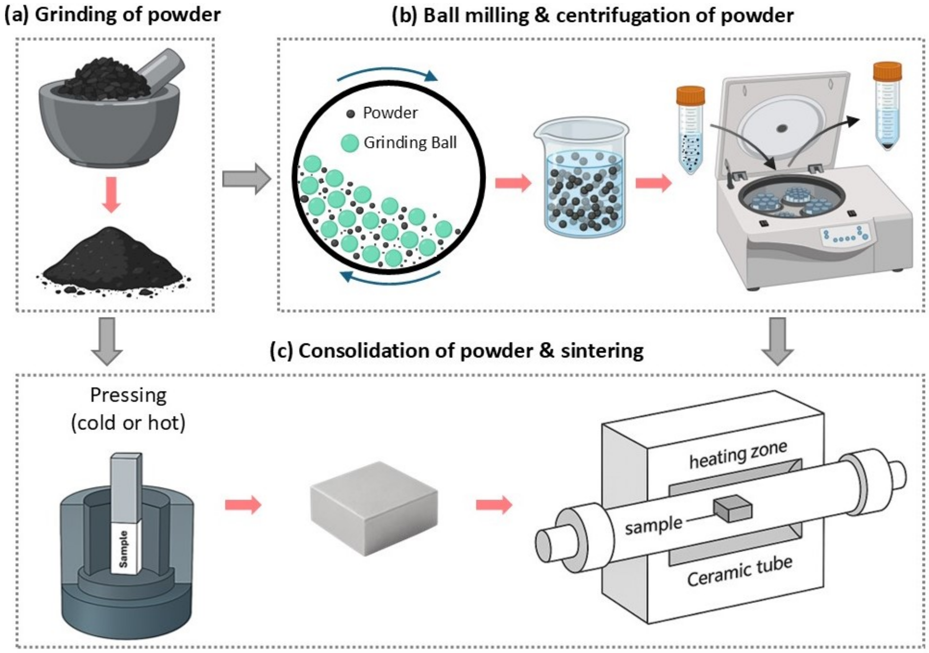

Figure S6. A schematic of $Bi_{0.3}Sb_{1.7}Te_3$ sample preparation: (a) manually grinding pieces of a $Bi_{0.3}Sb_{1.7}Te_3$ ingot in an agate mortar, (b) ball milling of the resulting $Bi_{0.3}Sb_{1.7}Te_3$ powder followed by centrifugation to classify powders into three fractions with average particle sizes of approximately 10.5 μm, 1.5 μm, and 200 nm, (c) placing each type of powder into a stainless steel die and consolidating via uniaxial pressing (cold or hot) followed by vacuum sintering to fabricate $Bi_{0.3}Sb_{1.7}Te_3$ bulk samples.

Data S7. Optimization of sintering temperature and time

To optimize the fabrication process, the effects of sintering temperature and duration on the TE properties were systematically investigated.

Effect of Sintering Temperature

Cold-pressed samples were sintered at different temperatures (593 K, 643 K, 693 K, and 743 K) for 5 h. As shown in Figure S7(a,b), increasing the sintering temperature from 593 K to 693 K led to a clear enhancement in σ while maintaining a relatively high S. In contrast, sintering at 743 K resulted in a dramatic increase in σ but a severe reduction in the S, particularly at room temperature. The *PF* reflects this trade-off. Although the 743 K sample exhibited the highest σ, its significantly reduced S led to inferior *PF* at room temperature. The sample sintered at 693 K for 5 h demonstrated the best balance between σ and S, resulting in the highest overall *PF* in the measured temperature range, as shown in Figure S7(c). Therefore, 693 K was identified as the optimal sintering temperature.

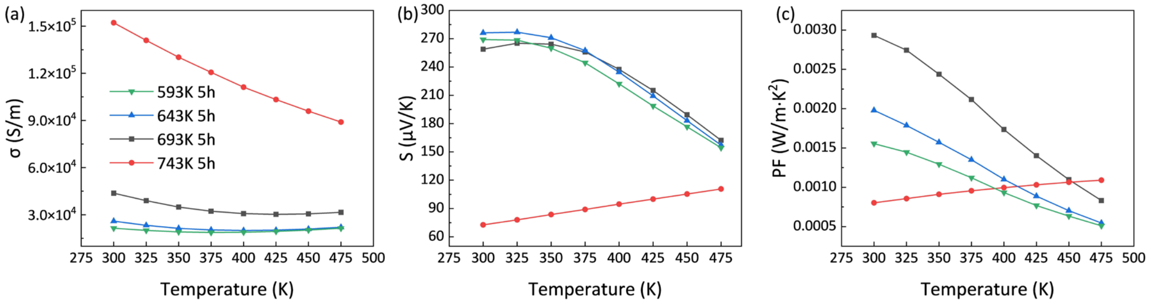

Figure S7. Sintering temperature optimization of $Bi_{0.3}Sb_{1.7}Te_3$ samples

Effect of Sintering Time at 693 K

Based on the temperature optimization, the sintering duration at 693 K was further varied (2 h, 5 h, and 8 h). As shown in Figure S8, extending the sintering time from 2 h to 5 h increased the σ substantially, while the S remained nearly unchanged (~260 μV K^{-1} at room temperature). Consequently, the *PF* improved significantly. However, further prolonging the sintering duration to 8 h reduced the σ and thus the *PF*. This decline is likely associated with Te volatilization, excessive grain coalescence, and increased defect density under prolonged thermal exposure.

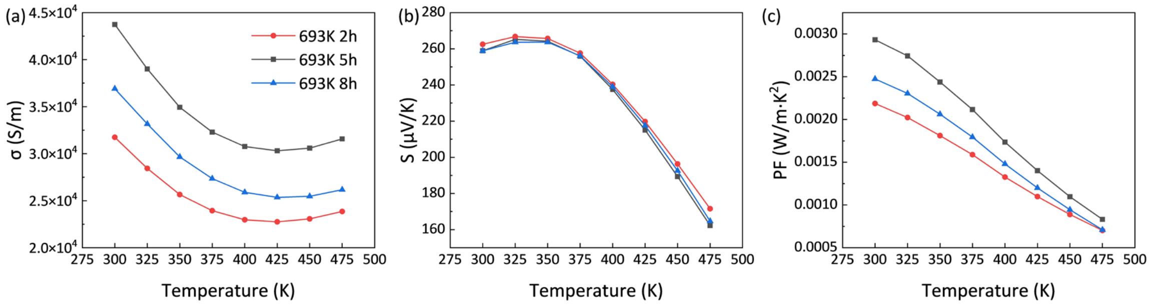

Figure S8. Sintering time optimization of $Bi_{0.3}Sb_{1.7}Te_3$ samples

Supplemental References:

[S1] Gibbs, Z. M., Kim, H.S., Wang, H., and Snyder, G. J. (2015). Band gap estimation from temperature dependent Seebeck measurement—Deviations from the 2e|S|maxTmax relation. Appl. Phys. Lett. 106, 022112. https://doi.org/10.1063/1.4905922

[S2] Bano, S., Kumar, A., Govind, B., Khan, A. H., Ashok, A., and Misra, D. K. (2020). Room temperature Bi2Te3-based thermoelectric materials with high performance. J. Mater. Sci-Mater. El. 31, 8607-8617. https://doi.org/10.1007/s10854-020-03396-6

[S3] Sun, Y., Wu, H., Dong, X., Xie, L., Liu, Z., Liu, R., Zhang, Q., Cai, W., Guo, F., and Sui, J. (2023). High Performance BiSbTe Alloy for Superior Thermoelectric Cooling. Adv. Funct. Mater. 33, 2301423. https://doi.org/10.1002/adfm.202301423

[S4] Hu, L.P., Zhu, T.J., Wang, Y.G., Xie, H.H., Xu, Z.J., and Zhao, X.B. (2014). Shifting up the optimum figure of merit of p-type bismuth telluride-based thermoelectric materials for power generation by suppressing intrinsic conduction. NPG Asia Mater. 6(2), e88-e88. https://doi.org/10.1038/am.2013.86

[S5] Zheng, Y., Zhang, Q., Su, X., Xie, H., Shu, S., Chen, T., Tan, G., Yan, Y., Tang, X., Uher, C., and Snyder, G. J. (2015). Mechanically Robust BiSbTe Alloys with Superior Thermoelectric Performance: A Case Study of Stable Hierarchical Nanostructured Thermoelectric Materials. Adv. Energy Mater. 5, 1401391. https://doi.org/10.1002/aenm.201401391

[S6] Madavali, B., Kim, H., and Hong, S.J. (2019). Reduction of thermal conductivity in Al2O3 dispersed p-type bismuth antimony telluride composites. Mater. Chem. Phys. 233, 9-15. https://doi.org/10.1016/j.matchemphys.2019.05.023.

[S7] Zhao, P., Yu, F., Wang, B., Zhao, H., Chen, C., Wang, D., Ying, P., Wu, Y., Li, P., Zhang, B., et al. (2021). Porous bismuth antimony telluride alloys with excellent thermoelectric and mechanical properties. J. Mater. Chem. A. 9, 4990-4999. https://doi.org/10.1039/D0TA09795K

[S8] Yang, G., Niu, R., Sang, L., Liao, X., Mitchell, D. R. G., Ye, N., Pei, J., Li, J.F., and Wang, X. (2020). Ultra-High Thermoelectric Performance in Bulk BiSbTe/Amorphous Boron Composites with Nano-Defect Architectures. Adv. Energy Mater. 10, 2000757. https://doi.org/10.1002/aenm.202000757

[S9] Madavali, B., Shin, D.W., Kim, D.S., and Hong, S.J. (2019). Enhanced thermoelectric properties by effective decoupling of electrical, thermal properties and seebeck coefficient through the addition of rare earth sesquioxides in p-type BiSbTe alloys. Intermetallics, 105, 139-145. https://doi.org/10.1016/j.intermet.2018.11.017

[S10] Li, J., Tan, Q., Li, J.F., Liu, D.W., Li, F., Li, Z.Y., Zou, M., and Wang, K. (2013). BiSbTe-Based Nanocomposites with High ZT: The Effect of SiC Nanodispersion on Thermoelectric Properties. Adv. Funct. Mater. 23, 4317-4323. https://doi.org/10.1002/adfm.201300146

[S11] Dharmaiah, P., Kim, D.H., Kwon, J.G., Lee, Y., Geum, S., Lee, G.R., Kang, M., and Hong, S.J. (2022). Optimization of mixed grain size structure for enhancement of thermoelectric figure of merit in p-type BiSbTe-based alloys. J. Mater. Sci. 57, 18131-18141. https://doi.org/10.1007/s10853-022-07532-x

[S12] Pakdel, A., Guo, Q., Nicolosi, V., and Mori, T. (2018). Enhanced thermoelectric performance of Bi–Sb–Te/Sb2O3 nanocomposites by energy filtering effect. J. Mater. Chem. A. 6, 21341-21349. https://doi.org/10.1039/C8TA08238C

[S13] Pakdel, A., Khan, A. U., Pawula, F., Hébert, S., and Mori, T. (2022). Effect of Bi Nanoprecipitates on the Thermoelectric Properties of Bi-Sb-Te/Sb2O3 Nanocomposites. Adv. Mater. Interfaces. 9, 2200785. https://doi.org/10.1002/admi.202200785

[S14] Li, C., Ma, S., Wei, P., Zhu, W., Nie, X., Sang, X., Sun, Z., Zhang, Q., and Zhao, W. (2020). Magnetism-induced huge enhancement of the room-temperature thermoelectric and cooling performance of p-type BiSbTe alloys. Energy Environ. Sci. 13, 535-544. https://doi.org/10.1039/C9EE03446C

[S15] Xing, L., Cui, W., Sang, X., Hu, F., Wei, P., Zhu, W., Nie, X., Zhang, Q., and Zhao, W. (2021). Enhanced thermoelectric performance and atomic-resolution interfacial structures in BiSbTe thermo-electro-magnetic nanocomposites incorporating magnetocaloric LaFeSi nanoparticles. J. Materiomics. 7, 998-1006. https://doi.org/10.1016/j.jmat.2021.02.013

[S16] Asai, J., Bumrungpon, M., Tsubochi, T., Kanaya, T., Tachii, M., Maeda, T., Iwamoto, T., Kanda, C., and Hasezaki, K. (2022). Experimental estimation of the Lorenz number and scattering parameter for p-type bismuth antimony telluride via multiple doping under constant temperature conditions. Ceram. Int., 48, 12520-12528. https://doi.org/10.1016/j.ceramint.2022.01.119

[S17] Hasezaki, K., Hamachiyo, T., Ashida, M., Ueda, T., and Noda, Y. (2010). Thermoelectric Properties and Scattering Factors of Finely Grained Bi2Te3 Related Materials Prepared by Mechanical Alloying. Mater. Trans. 51, 863-867. https://doi.org/10.2320/matertrans.MH200901

[S18] Satterthwaite, C. B., and Ure, R. W. (1957). Electrical and Thermal Properties of Bi2Te3. Phys. Rev. 108(5), 1164-1170. https://doi.org/10.1103/PhysRev.108.1164
